# Better together: novel methods for measuring and modeling development of executive function diversity while accounting for unity

**DOI:** 10.3389/fnhum.2023.1195013

**Published:** 2023-07-24

**Authors:** Jessica Wise Younger, Kristine D. O’Laughlin, Joaquin A. Anguera, Silvia A. Bunge, Emilio E. Ferrer, Fumiko Hoeft, Bruce D. McCandliss, Jyoti Mishra, Miriam Rosenberg-Lee, Adam Gazzaley, Melina R. Uncapher

**Affiliations:** ^1^Neuroscape, Department of Neurology, Weill Institute for Neurosciences, University of California, San Francisco, San Francisco, CA, United States; ^2^Department of Psychiatry, University of California, San Francisco, San Francisco, CA, United States; ^3^Department of Psychology & Helen Wills Neuroscience Institute, University of California, Berkeley, Berkeley, CA, United States; ^4^Department of Psychology, University of California, Davis, Davis, CA, United States; ^5^Department of Psychological Sciences and Brain Imaging Research Center (BIRC), University of Connecticut, Storrs, CT, United States; ^6^Graduate School of Education, Stanford University, Stanford, CA, United States; ^7^Department of Psychiatry, University of California San Diego, La Jolla, CA, United States; ^8^Neural Engineering & Translation Labs, University of California San Diego, La Jolla, CA, United States; ^9^Department of Psychology, Rutgers University, Newark, NJ, United States; ^10^Department of Psychiatry and Physiology, University of California, San Francisco, San Francisco, CA, United States; ^11^Advanced Education Research and Development Fund, Oakland, CA, United States

**Keywords:** executive function, in-school assessment, network modeling, middle childhood, digital assessment

## Abstract

**Introduction:**

Executive functions (EFs) are linked to positive outcomes across the lifespan. Yet, methodological challenges have prevented precise understanding of the developmental trajectory of their organization.

**Methods:**

We introduce novel methods to address challenges for both measuring and modeling EFs using an accelerated longitudinal design with a large, diverse sample of students in middle childhood (*N* = 1,286; ages 8 to 14). We used eight adaptive assessments hypothesized to measure three EFs, working memory, context monitoring, and interference resolution. We deployed adaptive assessments to equate EF challenge across ages and a data-driven, network analytic approach to reveal the evolving diversity of EFs while simultaneously accounting for their unity.

**Results and discussion:**

Using this methodological paradigm shift brought new precision and clarity to the development of these EFs, showing these eight tasks are organized into three stable components by age 10, but refinement of composition of these components continues through at least age 14.

## 1. Introduction

Executive functions (EFs) comprise a variety of cognitive abilities that enable agency over one’s attention (for review see e.g., Diamond, 2013; Zelazo et al., 2016). EFs are a critical set of skills as they consistently predict positive outcomes in school and across the lifespan (Moffitt et al., 2011; Schlam et al., 2013; Pascual et al., 2019; Spiegel et al., 2021). Thus, understanding how EFs emerge and change across development is critical to understanding how we might support their growth during periods of vulnerability and opportunity (from early childhood through and into adulthood). Like most complex cognitive processes, defining and measuring EFs has been complicated, and has not yielded a consistent taxonomy of EFs (see e.g., Morra et al., 2018). While a dominant conception of how EF is organized at least in adults (Miyake et al., 2000b) proposes that EFs comprise at least three components such as holding and working with information in mind (“working memory”), the flexibility to switch between multiple tasks, goals, or rules (“cognitive flexibility”), and the attentional or inhibitory control that allows one to focus on goal-relevant information while filtering out goal-irrelevant information (“attentional control”), even in their seminal 2000 paper, Miyake and colleagues suggested these were not the only EFs. To date though, the number of EFs remains undetermined; a review of the literature found as many as 18 EFs (Packwood et al., 2011). Yet, neural data suggest EF components are more alike than different, relying on similar networks, rather than operating as distinct, independent processes (see Niendam et al., 2012 for review). Indeed, the neuroimaging field has developed sophisticated methods for interrogating the complex, dynamic relationships between components of neural systems, yet, methods for measuring and modeling such dynamic interactions using behavioral input have lagged behind. Much of the developmental literature, for example, has examined components as separate constructs. We propose that to close this gap between models of neural and behavioral data, we must build on our methodological toolkit to enable the measurement and modeling of how cognitive processes, including EFs, function in concert to achieve a specific goal (Doebel, 2020). Here we introduce two novel methods, one for measurement and one for modeling, to understand how EFs manifest over development with data we collected in a large, accelerated longitudinal study with a diverse sample of students over two years. First, we show how a novel, adaptive EF assessment battery solves previous challenges to *measuring* EFs consistently. We then *model* these data using network analytic techniques to account for what is common across EFs to reveal a clear timeline of EF development during middle childhood, a particularly understudied period.

A fundamental question yet to be fully addressed by the field is how the various components of EFs are organized across development. In other words, are the three components described above the most accurate way to parse EF in both children and adults? Second, does this taxonomy of EFs change over development, and if so, when and in what way? A key developmental theory posits that EFs begin as a unitary construct in early childhood, and the differentiation of specialized components over time is initiated by experience, to become the multi-dimensional construct observed by young adulthood (Shing et al., 2010; Mungas et al., 2013). This *differentiation hypothesis* aligns with neural developmental evidence showing increased specialization of the neural systems supporting EFs (Johnson, 2011). Findings from developmental studies using latent factor analyses have been roughly consistent with the idea of increasing differentiation of EF components from preschool through adolescence (see Lee et al., 2013 for review). During middle childhood (approximately ages 7–13), reports of the number of factors of EF have varied between one (e.g., (Shing et al., 2010; Xu et al., 2013) and four (e.g., Agostino et al., 2010). However, to date the precise understanding of when individual components begin to differentiate remains unclear. Behavioral tests of this hypothesis to-date have largely almost all taken a latent variable approach, modeling EF components as related but distinct processes and failing to adequately account for the commonalities between components. Thus, despite decades of studies, there is not yet a clearly established pattern regarding the number of distinguishable components at each age. The lack of established developmental trajectories of EFs hinders progress in understanding how specific EFs might support various health and academic outcomes, and therefore how development of these skills might be supported and when in order to benefit student outcomes. The inconsistencies in the extant literature call for a paradigm shift in approach to both the measurement and modeling of EF performance to move beyond fragmented views of EF and toward treating them as a dynamic interconnected network of skills. Next, we outline the critical factors that could comprise such an approach and offer evidence in support of the promise of such an approach.

### 1.1. Measuring EFs

To reveal the developmental trajectory of EFs, we first need to measure EFs with assessments that are robust across developmental stages and assessment sessions. Much of the prior cross-sectional and longitudinal work has been confounded by (a) use of the *same* tasks across age ranges, which results in floor or ceiling effects in performance if the challenge level is not adjusted appropriately, or (b) use of *different* tasks with different age groups which prevents comparisons between groups (as reviewed in e.g., Best and Miller, 2010). Not only must tasks be comparable across age and ability, but EF assessments also need to be *repeatable* over multiple timepoints so developmental progress can be measured within subjects without practice or ceiling effects. Adaptive methods that use tasks that dynamically adjust to an individual’s appropriate challenge level on a trial-by-trial basis, presents a compelling and simple solution to this pernicious problem (Anguera et al., 2016b; Draheim et al., 2020). Indeed, prior work with pediatric populations suggests that highly engaging assessments with adapting challenge algorithms can reveal phenotypic differences between clinical and neurotypical populations, even when group characteristics are highly variable (Anguera et al., 2016a).

We further need *multidimensional assessments* to disentangle what EFs have in common from what they uniquely contribute to performance, to ensure each component is measured validly and reliably. Any single task used to assess a component of EF will necessarily involve processes not related to EFs (e.g., visual processing, motor response), or may be related to multiple EF components, both of which will result in measurement impurity (Miyake et al., 2000a; Diamond, 2013). To address this impurity, researchers can collect multiple measures of each hypothesized component of EF, leveraging the commonalities across tasks to extract information about EF skills, and reducing the contribution of idiosyncratic skill related to any individual task. Thus, methods that use multiple indicators to measure each hypothesized component of EF are critical for a robust and reliable understanding of how EFs develop over time.

Lastly, to understand how EFs are expressed in real-world contexts such as school or home, recent work suggests EFs should be assessed in real-world contexts (e.g., Anderson, 2002). Indeed, one study showed that while in-school EF assessments administered in group vs. individual contexts were highly correlated, only scores from group administered assessments uniquely predicted academic achievement (Obradović et al., 2018). A related study revealed that an individual’s growth in EF skills over the course of the school year can be influenced by classmates’ performance (Finch et al., 2019). Thus, examining EFs in real-world educational settings (in-school, group administered contexts) provides a more ecologically valid context and is thus a necessary strategy for understanding the veridical relation between EFs and academic achievement (as reviewed in McCoy, 2019).

Here we introduce a novel assessment tool—Adaptive Cognitive Evaluation Classroom (ACE-C)—that addresses these robust measurement needs. ACE-C is based on the original ACE battery described in Anguera et al. (2016b), modified for use with children and amenable to administration in large group settings. ACE-C is a battery of assessments that taps multiple EFs through several different tasks. Importantly, each task incorporates adaptive algorithms, allowing the repeated measurement of EFs across multiple timepoints, using the same tasks in different age groups without running into floor or ceiling limitations. The incorporation of adaptive algorithms across several different tasks represents a significant advancement in assessment capabilities in two significant ways. First, this work complements prior development of EF batteries that have been used across ages (e.g., NIH Toolbox; Zelazo and Bauer, 2013; Minnesota Executive Function Scale (MEFS); Carlson and Zelazo, 2014) by bringing additional dimensionality to the assessments, allowing for examination of individual EF components across individuals. Second, building off of methods that adjust task parameters at the population-level (e.g., Davidson et al., 2006), the adaptive algorithms in ACE-C operate at an individual level. As such, no assumptions are made about the individual before interacting with ACE-C, which ensures that even individuals who perform above or below what might be expected based on demographic variables (e.g., age or grade) receive the same experience as individuals on more typical developmental trajectories. Further, this individualized adjustment is done automatically, without additional input from the experimenter, which facilitates large-group assessment even across diverse groups of individuals.

### 1.2. Modeling EFs

Understanding the complexity of EF developmental trajectories requires not only solving measurement challenges, but also solving concomitant modeling challenges. Historically, latent variable analysis has been the most common approach to evaluating the changing organization of EFs over development (Karr et al., 2018). With latent variable analysis, we have come to understand that across the lifespan, while EFs diversify over development, they do not become completely distinct. Indeed, both behavioral and neural examinations of EFs have demonstrated the existence of a unifying umbrella construct termed “Common EF” through adulthood (Friedman et al., 2008; Reineberg et al., 2015; Friedman and Miyake, 2017; Smolker et al., 2018). Notably, cross-sectional examinations of middle childhood and adolescence using latent variable models also support the inclusion of a Common EF component (Engelhardt et al., 2015; Hatoum et al., 2020) as well. However, including such a factor to test the differentiation hypothesis and assess the dynamic development of EFs longitudinally poses severe methodological challenges.

While Common EF can be modeled from the confirmatory approach by incorporating it as a higher-order umbrella component capturing what is common among all lower-order components, such an approach is not amenable to testing the differentiation hypothesis. Models with a higher-order component would require at least three lower-order components of EF to be properly identified (Kline, 2011) and provide meaningful insight into the patterns of the behaviors being modeled. Thus a model with only one or two components differentiated from Common EF is not identified, and the earliest stages of differentiation cannot be examined. An alternative modeling approach is to incorporate Common EF not in a hierarchical fashion, but as an additional lower-order latent variable. In such a model, each observed variable measures two latent variables, Common EF and another differentiated component (a “bifactor” model). While these models can be easier to identify in some instances, it can be difficult to get such complex models to converge given the historically low power and task reliability observed in extant examinations of EF (Karr et al., 2018).

Further, confirmatory latent analyses provide limited information as to how the cognitive mechanisms supporting EF task performance may evolve over development (e.g., whether a task may index different EF components at different developmental stages). While model fit statistics can indicate whether a hypothesized organization fits the observed data well, they provide limited information on how to improve that model. For example, while one hypothesized organization might fit the data well, there could be other organizations that fit the data better that simply go untested. Additionally, methods for statistically comparing alternate hypotheses regarding which component a task measures are not straightforward. As such, alternative hypotheses around the EFs involved in different tasks are unlikely to be developed from the results of confirmatory latent analyses.

To advance our understanding of how EFs evolve over development, we need a method that (a) allows for task performance to reflect different EFs at different developmental stages, and (b) accounts for the high degree of association common to all EF tasks. Exploratory latent variable models like exploratory factor analysis (EFA) meet the first requirement but fail to account for Common EF. Conversely, confirmatory approaches such as confirmatory factor analysis (CFA) and bifactor modeling can account for commonalities among EF tasks but do not allow for task reorganization in a data-driven way. Indeed, recent evidence has suggested latent variable analysis may not be an appropriate representation of EFs (Camerota et al., 2020). To build on the insights gained from latent variable modeling, we suggest leveraging a powerful family of techniques that provides a data-driven method for identifying unique and communal cognitive mechanisms: network analysis. Network analysis is an approach gaining traction in the psychometric field for understanding cognitive constructs comprising complex inter-related components such as intelligence, psychopathology, and personality (Borsboom and Cramer, 2013; Costantini et al., 2015; Kan et al., 2019). In network analysis, relationships between variables can be determined after accounting for what is common among all variables by examining partial correlations. Thus, through network analysis, we can understand how EF behaviors are related after what is common among all variables, including what can be attributed to Common EF, is accounted for. Further, in contrast to latent variable analysis, in which observed variables are related through the modeled unobserved latent variables, the relationships between observed variables is direct. As such, performance on one task can affect performance on any other, not just those tasks theorized to measure the same construct. Finally, after determining how each variable is related to another, we can assess which variables likely reflect the same cognitive construct by applying community detection algorithms. This data-driven approach groups together variables that are more strongly related to each other than other variables in the network, allowing us to establish a theory-agnostic organization of EFs. In this way, network analysis allows us to examine the structure of EF from a holistic perspective and arrive at the organization that best reflects the data without testing and comparing multiple competing models.

### 1.3. Current study

Here, we capitalize on the improved interpretability of longitudinal and cross-sectional comparisons afforded by using the same tasks across all participants (Best and Miller, 2010) with our ACE-C battery to shine light on the relatively understudied period of middle childhood (∼7–12 years old), the developmental stage in which EFs may undergo the most rapid organizational development (Romine and Reynolds, 2005; Best et al., 2011). We demonstrate how network analysis can advance our understanding of the organization of three hypothesized EFs across development by first testing the differentiation hypothesis with the latent variable analysis approach and then highlighting the additional insights gained by using a network analysis approach. Specifically, we use each method to determine not only when the investigated EFs become distinct from one another but, critically, when they become distinct from the unifying Common EF component. Finally, we leverage information generated from network analyses to gain insights into the stability of the organization of these EFs across time. We show that during middle childhood, organization of these EFs begins to stabilize, yet continues to develop in a manner suggesting EFs need continued support throughout their protracted development as children transition to adolescence. Developmental insights revealed by network analyses extend those from latent variable analyses and, in line with work by Camerota et al. (2020), show how differing modeling methods can result in different conclusions regarding the number of components identified across development to date. The novel findings from network analysis lay the groundwork for new avenues of investigation to understand how to best support EFs across the lifespan.

## 2. Materials and methods

Participants in this study were recruited through their schools as part of Project iLEAD (in-school longitudinal executive function and academic achievement database), a two-year accelerated longitudinal study of EF development in students grades 3–8. Full details of Project iLEAD are reported in Younger et al. (2022).

The study was approved by the University of California San Francisco Institutional Review Board and conducted in accordance with the relevant guidelines and regulations. Written parental or guardian consent was obtained from all participants at the beginning of the study, and verbal assent from all participants was obtained before all in-class data collection sessions. At the end of the study, all students in participating classrooms received snacks and stickers, regardless of participation.

### 2.1. Participants

Nine schools (seven public, one independent, and one parochial) from northern California opted to participate in this longitudinal study, which included assessments at the Fall and Spring of two academic years for a total of four assessment periods. Two of the five public elementary schools and one of the two public middle schools were Title I schools. In total, 1,280 students participated over the course of two years. At the beginning of each school year, teachers distributed consent forms to students to take home for parental or guardian review and signature. This first round of recruitment resulted in a total of 1,088 participating students in Year 1: 284 3rd graders (*M* = 8.07 years old, *SD* = 0.35), 260 5th graders (*M* = 9.98 years old, *SD* = 0.41), and 544 7th graders (*M* = 11.9 years old, *SD* = 0.47). In the fall of Year 2, we re-opened enrollment for participating classrooms to allow new students to participate in the study, which resulted in an additional 195 students joining the study (44 4th, 147 6th, and 4 8th grade students). The Year 2 sample thus included 1,106 students: 288 4th graders (*M* = 9.03 years old, *SD* = 0.33), 336 6th graders (*M* = 10.9 years old, *SD* = 0.39), and 482 8th graders (*M* = 12.9 years old, *SD* = 0.44). For patterns of missing data across timepoints, see Supplementary Figure 1. Our sample was demographically diverse. Ethnically, our sample was 34% Asian, 26% Hispanic/Latinx, and 16% White. Further, 10% of the sample received Special Education services, 32% qualified as low income, and 14% were currently enrolled in English Language classes, with another 29% having previously been enrolled in English Language classes, but now considered fluent in English. See Table 1 for additional demographics of participating students.

**TABLE 1:** Student demographics.

		Timepoint 1		Timepoint 2		Timepoint 3		Timepoint 4	
		*n*	%	*n*	%	*n*	%	*n*	%
Gender	Male	519	50.9	514	51.8	541	51.3	510	51.2
	Female	500	49.1	478	48.2	513	48.7	487	48.8
Ethnicity	American Indian or Native Alaskan	5	0.5	6	0.6	6	0.6	5	0.5
	Asian	339	33.3	332	33.5	369	35.0	350	35.1
	Black or African American	20	2.0	18	1.8	18	1.7	17	1.7
	Filipino	55	5.4	56	5.7	60	5.7	56	5.6
	Hispanic or Latinx	267	26.2	253	25.5	280	26.6	269	27.0
	Pacific Islander	7	0.7	6	0.6	5	0.5	6	0.6
	Two or more ethnicities	43	4.2	45	4.5	44	4.2	44	4.4
	White	170	16.7	165	16.6	185	17.6	169	17.0
	Unknown	113	11.1	111	11.2	87	8.26	81	8.1
Special education status	No	806	79.1	771	77.7	855	81.1	807	80.9
	Yes	101	9.9	111	11.2	113	10.7	111	11.1
	Unknown	112	11.0	110	11.1	86	8.2	79	7.9
Low income qualification	No	583	57.2	570	57.5	616	58.4	587	58.9
	Yes	324	31.8	312	31.5	352	33.4	331	33.2
	Unknown	112	11.0	110	11.1	86	8.2	79	7.9
English language fluency	English monolingual	396	38.9	391	39.4	434	41.2	413	41.4
	English multilingual, never enrolled in English classes	56	5.5	58	5.9	67	6.4	63	6.3
	English multilingual, previously enrolled in English classes	308	38.9	289	29.1	324	30.7	311	31.2
	Current English Language Learner	147	14.4	144	14.5	143	13.6	131	13.1
	Unknown	112	11.0	110	11.1	86	8.2	79	7.9

### 2.2. Procedures

We administered a series of mobile assessments of EF, math, and reading skills that took the form of digital “games”, during school hours, at the beginning and end of each academic year (fall and spring) over two school years. At each of the four timepoints, EF assessments were administered during one class period (approximately 50 min), with the research team returning a little over a month later to administer the math and reading assessments (*M* = 5.7 weeks, *SD* = 2.4, min. = 1.9, max. = 10). At the end of each academic year, academic performance and other relevant data were provided by the district for students whose parents consented to share district data.

#### 2.2.1. Adaptive cognitive evaluation classroom (ACE-C)

This study used a novel mobile assessment battery, ACE-C, to assess EF skills. The original ACE battery was developed from cognitive assessments commonly used in lab-based settings and modified for real-world settings by including adaptive, psychometric staircase algorithms, highly motivating trial-wise and end-of-task feedback (Anguera et al., 2016b). ACE-C is an adaptation of this battery to include a child-friendly interface and additional instructional design to facilitate group-administration. Importantly, the adaptive algorithms enabled two critical affordances: (a) the same tasks could be used with the same students across multiple timepoints to reveal a student’s changing cognitive abilities without being confounded by ceiling or floor effects or reduced motivation due to multiple assessments, and (b) the same tasks could be used across students of diverse ages to reveal individual differences in cognitive abilities across development without the confound of different tasks (Anguera et al., 2016b). This advancement in our approach to assessment enabled robust integrative data analytics within-subjects over time, and across-subjects from a wide age range without any a *priori* assumptions about any individual participant’s abilities, for example, according to their age.

The assessment battery consisted of a color blindness test, a response time control task, two working memory tasks, one attentional filtering task, three context monitoring tasks, three interference resolution tasks, and one cognitive flexibility task. The attentional filtering task was excluded from the current analysis due to differential task challenge across grade levels, while the cognitive flexibility task was excluded due to technical errors that prevented consistent data reporting across timepoints. All other tasks are briefly described below along with the *a priori* defined metric of interest selected based on the psychometrics of each task. Full descriptions of each task are included in the Supplementary material. Example stimuli and schematics for tasks are presented in Supplementary Figure 2.

##### 2.2.1.1. Response time control task

The first ACE-C task was a measure of basic response time (*BRT*; Supplementary Figure 2A). Because improvements in EFs have also been associated with improvements in general processing speed (e.g., Fry and Hale, 1996), *BRT* was designed to serve as a covariate to be regressed from performance metrics of all other ACE-C tasks. By using BRT as a control metric, analyses were kept consistent across tasks and task-specific control metrics were not required. Mean response time (RT) collapsed across both dominant and non-dominant hands was the metric of interest for this task.

##### 2.2.1.2. Color blindness test

The second ACE-C task was a screening assessment for red-green color blindness (Ishihara, 1972; Supplementary Figure 2B). We assessed whether students selected one or more responses indicating red-green color blindness according to scoring guidelines in Ishihara (1972).

##### 2.2.1.3. Working memory

Two tasks were used to measure working memory (WM), *Forward Spatial Span* (Supplementary Figure 2C) and *Backward Spatial Span* (Supplementary Figure 2D). These two tasks were digital modifications based on the Corsi Block Task (Corsi, 1973). In this task, students were shown an array of open circles, with a target sequence cued via circles becoming filled, in sequence, with either green (*Forward Spatial Span*) or blue (*Backward Spatial Span*) color. Once students viewed the cued sequence, they were instructed to recreate the sequence in the same order (*Forward Spatial Span*) or in the reverse order (*Backward Spatial Span*). Sequence length increased according to performance. The metric of interest for both tasks was span length, or the maximum number of spatial locations attempted to be held in mind in the correct sequence.

##### 2.2.1.4. Context monitoring

Context monitoring (CM) was measured with three tasks: Sustained and Impulsive Attention (both tasks administered within a single test called *Continuous Performance Task* [*CPT*]*;* Supplementary Figure 2E) and *Tap and Trace* (Supplementary Figure 2F). For all three tasks, students were instructed to respond to a target stimulus and withhold a response to non-target stimuli. *CPT* is a target detection test adapted from the Test of Variables of Attention (TOVA; Greenberg et al., 1991). This test included two tasks: a target frequent task (80% target trials) designed to assess impulse control *(Impulsive Attention)* and a target infrequent task (20% target trials) designed to test sustained attention abilities *(Sustained Attention)*. For *Sustained Attention*, we used a metric that is sensitive to lapses in attention—the standard deviation of the RT to infrequently presented targets (Leark et al., 2018). For *Impulsive Attention*, we used a metric that would measure detection of targets while accounting for withholding prepotent responses to frequent non-targets–the signal detection metric of *d’*. *Tap and Trace* is a dual-task assessment adapted from the paradigm described by Eversheim and Bock (2001). This task included three blocked conditions: one in which students used their dominant hand to tap when they detected a target stimulus, a second in which they traced a shape with their non-dominant hand, and a third in which they performed both tasks simultaneously. To differentiate this task from the CPT and better address task impurity concerns by assessing context monitoring when EFs are challenged by divided attention, we included performance only on the dual-task block. For this task, the metric of interest was how reliably students could detect a target vs. a distractor during the dual-task portion of the task; thus, we again deployed *d’*.

##### 2.2.1.5. Interference resolution

Interference resolution (IR) was measured with three tasks: *Stroop* (Supplementary Figure 2G), *Flanker* (Supplementary Figure 2H), and *Boxed* (Supplementary Figure 2I). *Stroop* is based on the computerized version of the color-word *Stroop* task as described by Mead et al. (2002) in which students selected the text color (e.g., green) of a centrally presented color word (e.g., BLUE). On 30% of trials, the text and word were incongruent, and on 70% of trials they were congruent. *Flanker* is a letter flanker task based on the paradigm described by Eriksen and Eriksen (1974) in which students are instructed to indicate the middle letter of a string of five letters. On 50% of trials, the central and flanking letters were congruent, and on 50% of trials they were incongruent. Finally, *Boxed* is a top-down/bottom-up attention task based on the visual search paradigm first described by Treisman and Gelade (1980) in which students must identify a target stimulus in an array of distractor stimuli. This task included four blocked conditions that varied on search condition and number of distractors. In each condition, the target was either identifiable by one feature (color) or by the conjunction of two features (color of target and location of opening of the target box) and either a low (3) or high (11) number of distractor stimuli. For tasks in which students were expected to respond to each trial, we used Rate Correct Score (RCS) to index performance on both RT and accuracy. Task-level RCS was calculated by dividing the number of correct responses by the product of mean RT for all trials and the total number of trials responded to Woltz and Was (2006), Vandierendonck (2017) across all conditions. To achieve a high RCS, participants must perform quickly and accurately across all trials, regardless of condition. This approach thus takes into account how participants perform on both congruent and incongruent conditions without introducing reliability issues frequently cited when using more traditional subtraction methods (Enkavi et al., 2019). RCS was used for *Stroop* and *Flanker*, however, a technical error in *Boxed* prevented RCS from being calculated in the same manner as the other tasks. Instead, we used mean RT to all correct trials for *Boxed*. The grouping of tasks into these three components differs slightly from some extant literature in an effort to bring greater precision to the EFs measured. For extended discussion on the battery design and component grouping, see the “4. Discussion” section.

### 2.3. Analysis methods

#### 2.3.1. Data cleaning procedures

A very small number of students who were red-green colorblind as indicated by the colorblind screener (*n* = 16) were excluded from analysis, given that several tasks required students to discriminate between colors. Trials with no response when a response was expected and anticipatory trials (RT < 200 ms) were excluded from analyses (1.8% of all trials).

Data from each student were evaluated and cleaned on a task-level basis at each timepoint. In this way, participants were not wholly excluded from analysis, but only task data for which we could not be confident that the participant understood or complied with the task instructions were excluded. For each task, to be included in data analysis, students must have answered a minimum of five trials per condition and achieved above-chance accuracy on the easiest condition (i.e., the condition that required lowest cognitive load). Data from each task were then evaluated for outlier students based on performance within each cohort and timepoint. Outlier performance was defined as performance falling outside three median absolute deviations (MADs) of the median performance of the relevant cohort at a given timepoint (Leys et al., 2013). Finally, additional outlier analyses to identify influential observations in the larger regression analysis of task performance were conducted by computing Cook’s distance. Observations with Cook’s d > 1 were removed. These cleaning procedures resulted in exclusion of 1.9% of task-level data collected. See Supplementary Table 1 for *N* datasets excluded per task per cleaning step. For patterns of missing data across timepoints, see Supplementary Figure 2.

#### 2.3.2. Effects of age and time on task performance

For each task’s metric of interest, we sought to understand the developmental trajectory of performance across different age ranges. We used linear mixed effects models to examine how an individual student’s performance over time may differ depending on age after controlling for multiple demographic variables. To index the variable of time more precisely, it was coded as the number of months since last assessment. In this way, the first instance of a participant’s engagement with ACE-C was always indexed as 0, regardless of whether that occurred during the first timepoint of the study Fall 2016 or later (due to later enrollment, absence on data collection day, etc.). Age was indexed as participant age in months at the time of assessment. Control variables in these models included mean RT on the BRT task (continuous) as an indicator of general processing speed, cohort (3 categories: 3rd–4th grade, 5th–6th grade, 7th–8th grade), and gender (2 categories; male, female). Random effects included school (9 categories), the random intercept of participant, and the random slope of time. Models were run using the “lme4” package in R (Bates et al., 2014) and significance of each variable was evaluated using Satterthwaite’s degree of freedom method as implemented in the “lmerTest” package in R (Kuznetsova et al., 2017).

#### 2.3.3. Confirmatory factor analysis

We conducted latent variable modeling using confirmatory factor analysis (CFA). We chose to use CFA over exploratory factor analysis (EFA) because, while data-driven organizations of variables are possible using EFA, exploratory approaches do not provide a straightforward way to account for the high degree of overlap between performance on EF tasks, and assignment of a behavior to a latent variable is dubious, often resulting in uninterpretable organizations (Brocki and Bohlin, 2004). We conducted separate CFAs for the three cohorts at the four timepoints to avoid assuming the structure of EF remained the same across timepoints and to assess the stability of these structures over a two-year measurement period. We evaluated five models of EF: the maximally differentiated structure with three distinct factors, all possible permutations of a two-factor model in which two of the three factors are collapsed into one, and the simplest structure in which all tasks represent a single, undifferentiated EF factor (see Supplementary Figure 3). Although the longitudinal stability of models can be tested with a CFA approach, such statistical tests for longitudinal network analysis have not yet been developed. To keep the results of the two modeling techniques comparable, we do not account for the dependencies in observations across timepoints.

After assessing covariance coverage to ensure sufficient available data for all tasks across all cohorts and timepoints, all CFAs were conducted in Mplus version 8.1 (Muthén and Muthén, 2017) with the robust maximum likelihood estimation method. To statistically compare nested models, we used Satorra-Bentler scaled chi-square tests with degrees of freedom equal to the difference in number of free parameters between the comparison and nested models (Satorra and Bentler, 2010). These tests help us to determine whether more complex representations of EF are a better fit to EF task performance across middle childhood. Because these statistics are meant to compare nested models, the 1-factor model was compared to each of the 2-factor models, and each of the 2-factor models were compared to the 3-factor model, but the 2-factor models cannot be statistically compared to each other in this manner. In interpreting these results, we took a conservative approach in which a more complex model would be selected over a less complex model only if a more complex model would always be preferred, regardless of which 2-factor permutation was considered. The results of chi-square difference testing were corroborated by converging evidence from the Comparative Fit Index (CFI), root mean square error of approximation (RMSEA), Akaike Information Criteria (AIC), and sample-size adjusted Bayesian Information Criterion (BICc). CFI values > 0.90 were considered excellent model fit, with values closer to 1 indicating better model fit. RMSEA values less than or equal to 0.06 were considered adequate model fit (Hu and Bentler, 1999), with lower values indicative of better model fit.

Models explicitly incorporating a Common EF factor were not tested here, as models in which Common EF is a higher-order factor are not amenable to testing the differentiation hypothesis. While Common EF could be incorporated as a higher-order umbrella component reflecting what is common among all lower-order components, structures with any fewer than three differentiated components would not be considered properly identified (i.e., it would not be possible to uniquely estimate each component’s association with Common EF). While it is possible to test the differentiation hypothesis with an alternative approach incorporating Common EF as an additional lower-order latent variable, taking such an approach was not possible with our dataset. In such “bifactor” models, each observed variable measures two latent variables: Common EF and another differentiated component. Such a model would not be identified for this dataset without assuming performance on the WM tasks contributes equally to both the WM and Common EF factors (see Limitations), which has not been supported in the literature (Friedman et al., 2008, 2011).

#### 2.3.4. Network analysis

Replicating the general approach used for the latent variable models, we created separate models of EF performance for each cohort and timepoint. All network analyses were conducted in R 4.1.2 (R Core Team, 2020). Network models were estimated using the bootnet package (Epskamp, 2015). All models were fully saturated partial correlation networks (non-regularized Gaussian Markov random fields), and missing data were handled via full information maximum likelihood. After estimating each network model, the Spinglass algorithm (Reichardt and Bornholdt, 2006) from the igraph package (Csardi and Nepusz, 2006) was applied separately to each network to determine communities of tasks. We employed the Spinglass algorithm rather than other community detection algorithms, such as the Louvain algorithm, because it can handle negative partial correlations in a network. To ensure the stability of groupings, community detection was performed 1,000 times and the most frequent grouping is reported here. Resulting network and community detection results were displayed graphically using the qgraph package (Epskamp et al., 2012). For graphing purposes, nodes were fixed to the same positions across networks and partial correlations between -0.1 and 0.1 are not displayed. To understand network stability over time, edge weights from each network were correlated with each other. Because these edge weights represent partial correlations, edge weights were first Fischer transformed before computing correlations between networks.

## 3. Results

We first show how the use of novel, adaptive assessments can robustly measure EFs longitudinally across a wide age range without floor and ceiling effects. We then demonstrate how a holistic modeling approach that accounts for Common EF can enhance our current understanding of the emergence and development of EFs by testing the differentiation hypothesis using two analytic approaches, latent variable analysis and network analysis. Using a latent variable approach, we replicate the ambiguous, difficult to interpret results found in prior investigations. We then critically extend our understanding using a network analytic approach, revealing developmental insights missed under the latent variable approach that could not appropriately take into account Common EF.

### 3.1. Novel EF measurement

To examine the utility of our novel adaptive assessment, we performed two analyses, one to assess task performance, and another to assess challenge level. We had different predictions for each analysis. We predicted the adaptive response window would equate task challenge level across cohorts and timepoints as supported by similar percent of responses for which participants received “correct” feedback across cohorts and timepoints. However, we expected that task performance as measured by the metric of interest for each task noted above, which did not take into account whether the response was within the adaptive response window and may have included other aspects of performance such as response time (e.g., RCS, standard deviation of response time, d’, etc.), would show traditional developmental improvements in performance over time.

To confirm the effectiveness of the adaptive response window across tasks, we examined percent of responses with “correct” feedback only. In tasks with an adaptive response window (Impulsive Attention, Sustained Attention, Tap and Trace, Stroop, Flanker, and Boxed), participants only received “correct” feedback if they provided the correct answer within a limited time frame. All other responses resulted in feedback indicating the response was correct but “late” or “incorrect”. This adaptive algorithm was designed to produce ∼75% of responses resulting in “correct” feedback for all participants and while this target accuracy was not achieved across all tasks, it was confirmed in practice to produce an average of 72.04% across tasks. Additionally, the adaptive algorithm did not completely eliminate developmental effects; while linear models examining the effect of cohort and time on percentage of trials with “correct” feedback did show significant differences between cohort and timepoint However, the significance of these effects is likely driven by the large sample size used in the current study; model effect sizes were small, accounting for less than 20% of the variance across all tasks (average *R*^2^ = 0.10 see Table 2 and Supplementary Figure 5). Together, these results suggest the adaptive tasks successfully presented a similar challenge across ages and measurement occasions.

**TABLE 2:** Mean (standard deviation) percent of responses that received correct for tasks with an adaptive response window and variance explained by model of cohort and time.

	3rd–4th grade cohort				5th–6th grade cohort				7th–8th grade cohort				Model F (NumDF, DenDF)	Model R^2^	95% confidence interval
Timepoint	1	2	3	4	1	2	3	4	1	2	3	4			
Sustained attention	87.4 (6.6)	87.7 (5.6)	88.0 (6.8)	88.6 (5.2)	89.9 (3.2)	89.5 (3.3)	90.1 (4.3)	89.4 (5.2)	90.4 (3.5)	90.4 (3.1)	90.4 (5.7)	89.4 (7.0)	44.75 (3, 3918)	0.033	0.023, 0.045
Impulsive attention	73.9 (4.3)	74.2 (5.2)	75.1 (5.0)	75.0 (4.9)	76.0 (4.8)	75.9 (4.2)	76.0 (4.2)	76.2 (4.0)	76.9 (4.0)	77.0 (3.9)	76.8 (4.3)	76.6 (4.4)	78.06 (3,3954)	0.056	0.043, 0.071
Tap & trace	68.6 (8.3)	71.7 (8.4)	72.7 (8.7)	74.0 (9.0)	74.2 (7.4)	75.2 (7.7)	75.5 (8.4)	77.2 (8.2)	78.3 (6.0)	78.3 (8.1)	78.0 (8.0)	78.8 (6.6)	153.38 (3, 3049)	0.131	0.111, 0.154
Stroop	72.0 (6.7)	61.3 (9.5)	61.9 (9.9)	60.9 (9.7)	72.7 (5.3)	59.4 (9.3)	59.3 (9.7)	58.6 (9.5)	71.3 (5.3)	57.1 (10.0)	56.6 (9.4)	58.1 (9.0)	304.90 (3,3800)	0.194	0.173, 0.216
Flanker	66.8 (7.2)	64.1 (7.6)	63.9 (6.8)	63.3 (6.8)	68.7 (6.8)	63.3 (6.5)	63.7 (7.0)	62.3 (6.0)	69.3 (5.2)	62.2 (7.4)	62.2 (7.0)	62.3 (7.3)	111.86 (3, 3267)	0.093	0.076, 0.113
Boxed	62.2 (4.4)	63.0 (4.6)	64.0 (4.3)	64.3 (3.8)	64.3 (4.0)	65.4 (3.5)	64.9 (3.9)	65.7 (3.8)	65.2 (4.2)	65.6 (3.9)	66.2 (3.7)	66.1 (3.7)	95.51 (3, 3755)	0.071	0.057, 0.088

Data are collapsed across the four timepoints. NumDF, numerator degrees of freedom; DenDF, denominator degrees of freedom.

**FIGURE 1:**
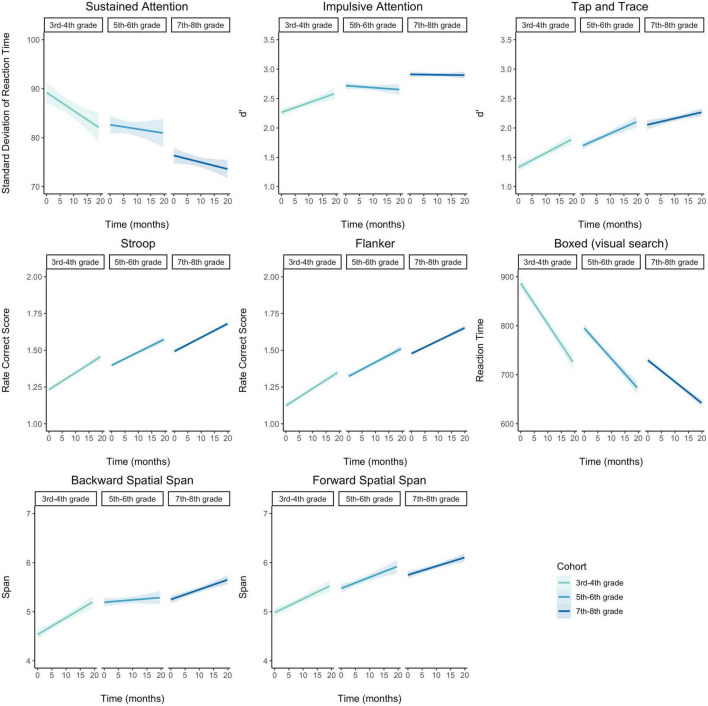
Growth in performance on executive function metrics of interest for each task and cohort. With few exceptions, all participants improved over time, and younger students tended to show the most gains over time, as indicated by significant main effects. Shaded region represents 95% confidence interval of linear regression of time on performance.

We next examined the potential developmental effect on task performance as measured by the task-specific metric of interest described above which did not take into account whether the response was within the adaptive response window and may have included other aspects of performance such as response time (e.g., RCS, standard deviation of response time, d’, etc.). We found each adaptive EF assessment captured predicted developmental improvements in performance. Linear mixed effects models examining task performance for each metric of interest allowing random effects for participant, school, and time, showed that, across tasks, performance significantly improved with age and time after controlling for BRT, cohort, and gender except the two span tasks. Both Forward and Backward Spatial Span showed significant effects of time, but only trended towards main effects of age, possibly due to the ordinal nature of the metric of task performance for these tasks which leaves little room for variation. Plots of raw scores not accounting for these control variables are shown in Figure 1 and effect sizes of each control variable are shown in Supplementary Figure 4. For between task correlations as well as the mean and standard deviation of task performance after accounting for BRT for each cohort and timepoint (the metric used in modeling analyses), see Supplementary Tables 2–4. Beyond these predicted EF performance improvements with age, performance on all but two tasks (Tap and Trace and Backwards Spatial Span) showed a significant interaction between age and time, suggesting that younger participants tended to improve more over time compared to older participants. Across tasks, the two control variables that most frequently had a significant effect on performance were BRT and gender. The consistently strong effect of BRT on all tasks was expected as this variable was included to capture potential differences in an individual’s pattern of responses, which might also capture such variance due to familiarity with responding on a touch-screen device, etc. Further, for all but Sustained Attention and Forward Spatial Span, there was a significant effect of gender, with students self-identifying as female showing better task performance compared to those identifying as male.

### 3.2. Novel EF modeling

After solving for persistent challenges to measuring EFs through the use of our novel tool, ACE-C, we demonstrate how network analysis can build on the findings from latent variable analysis and generate new hypotheses regarding the organization of EFs by accounting for what is common between EF components.

#### 3.2.1. Latent variable analysis

To directly test the differentiation hypothesis using latent variable modeling, we compared a series of models to establish the number of distinguishable EF components at each stage of development using CFA. In accordance with the differentiation hypothesis, we expected more complex models with more unique factors would provide better model fit for older students. Based on prior adult literature and the tasks used in the current study, the number of components could range from one to three, with the maximally-differentiated organization of EFs representing WM, IR, and CM grouping components. As noted in the methods, we did not explicitly incorporate Common EF into these models and instead examined correlations between factors to assess when these components could be differentiated beyond the unifying Common EF factor. Correlations greater than 0.70 between factors indicate that components represent redundant information (sharing more than 49% of variance) and are therefore likely not fully differentiated from one another.

Overall, the latent variable approach revealed an indeterminate developmental progression of differentiation of EF components. Model fit statistics (Supplementary Table 5) tended to indicate a 2-factor model was the best fitting model for the 3rd–4th grade cohort at all timepoints, though a different 2-factor model was the best fitting at each timepoint. At timepoint 1, the model with WM as distinct fit best, the model with IR as distinct fit best at timepoints 2 and 3, and the model with CM as distinct fit best at timepoint 4. However, it should be noted that at timepoints 1, 2, and 4, a 3-factor solution had similar fit statistics to these 2-factor solutions. Fit statistics were similarly mixed at timepoint 1 for the 5th–6th grade cohort, with both the 2-factor model in which WM is distinct and the 3-factor model showing best fit statistics. After timepoint 1 though, fit statistics pointed towards the 3-factor solution being the best fit through timepoint 4 for the 7th–8th grade cohort. However, statistical comparisons of the models indicated that while more complex models may have better fit indices, they may not be necessary to model the data well.

Generally, results of statistical comparisons (Table 3) suggest that a single component best describes the organization of EF from 3rd through 4th grade, after which at least three distinct EF components can be identified. However, this pattern is not unequivocal, and many open questions remain. Within the 3rd–4th grade cohort, at least two out of three 2-factor models did not provide significantly better model fit than a 1-factor model with the exception of timepoint 2. At this timepoint, even the 3-factor model provided better fit than all but the 2-factor model in which IR is distinct. Yet, at timepoint 3, a more complex model never provided significantly better fit compared to a single component, leaving the developmental trajectory unclear. Further, at the first timepoint for both the 5th–6th and 7th–8th grade cohort, the 2-factor model combining CM and IR fit significantly better than a single-factor model, but other potential 2-factor configurations did not fit the data better than models with a single component. Additionally, the 3-factor model did not fit better than the WM-distinct 2-factor model, indicating EFs may not be well-differentiated at timepoint 1 for any age group. Moreover, alternative hypotheses around the EFs involved in different tasks are unlikely to be developed from these results. Different structures from those tested here may fit the data better (e.g., a task may index a different EF component at different developmental stages), but methods for statistically comparing such alternate hypotheses regarding which EF component a task draws on are not straightforward and would not be feasible to test without additional theoretical guidance.

**TABLE 3:** Satorra–Bentler scaled χ^2^ tests comparing 1-, 2-, and 3-factor models of executive function.

			1- versus 2-Factor (IR with CM)		1- versus 2-Factor (WM with CM)		1- versus 2-Factor (WM with IR)		2- (IR with CM) versus 3-Factor		2- (WM with CM) versus 3-Factor		2- (WM with IR) versus 3-Factor	
Cohort	Time- point	*n*	Δχ^2^(Δd*f*)	*p*	Δχ^2^(Δd*f*)	*p*	Δχ^2^(Δd*f*)	*p*	Δχ^2^(Δd*f*)	*p*	Δχ^2^(Δd*f*)	*p*	Δχ^2^(Δd*f*)	*p*
3rd-4th grade cohort	1	210	10.602 (1)	**0.001**	0.157 (1)	0.692	0.225 (1)	0.635	0.277 (2)	0.871	10.642 (2)	**0.005**	11.199 (2)	**0.004**
	2	209	4.539 (1)	**0.033**	28.671 (1)	**<0.001**	20.036 (1)	**<0.001**	21.504 (2)	**<0.001**	3.486 (2)	0.175	12.052 (2)	**0.002**
	3	217	0.002 (1)	0.962	2.975 (1)	0.085	1.449 (1)	0.229	3.662 (2)	0.160	0.255 (2)	0.880	2.155 (2)	0.340
	4	234	2.804 (1)	0.094	3.513 (1)	0.061	11.037 (1)	**0.001**	10.544 (2)	**0.005**	10.116 (2)	**0.006**	2.397 (2)	0.302
5th–6th grade cohort	1	211	9.077 (1)	**0.003**	1.5 (1)	0.221	3.059 (1)	0.080	3.056 (2)	0.217	10.232 (2)	**0.006**	9.389 (2)	**0.009**
	2	201	9.685 (1)	**0.002**	18.086 (1)	**<0.001**	9.551 (1)	**0.002**	12.839 (2)	**0.002**	7.828 (2)	**0.020**	14.254 (2)	**0.001**
	3	281	10.905 (1)	**0.001**	2.194 (1)	0.139	17.954 (1)	**<0.001**	15.696 (2)	**<0.001**	30.375 (2)	**<0.001**	10.741 (2)	**0.005**
	4	273	9.23 (1)	**0.002**	14.339 (1)	**<0.001**	38.936 (1)	**<0.001**	18.365 (2)	**<0.001**	13.323 (2)	**0.001**	7.003 (2)	**0.030**
7th–8th grade cohort	1	447	10.761 (1)	**0.001**	2.335 (1)	0.126	5.982 (1)	**0.014**	5.445 (2)	0.066	14.184 (2)	**0.001**	10.575 (2)	**0.005**
	2	453	13.883 (1)	**<0.001**	5.112 (1)	**0.024**	6.9 (1)	**0.009**	6.739 (2)	**0.034**	15.111 (2)	**0.001**	13.203 (2)	**0.001**
	3	432	26.161 (1)	**<0.001**	7.093 (1)	**0.008**	21.051 (1)	**<0.001**	18.276 (2)	**<0.001**	38.066 (2)	**<0.001**	20.456 (2)	**<0.001**
	4	410	42.235 (1)	**<0.001**	20.249 (1)	**<0.001**	37.355 (1)	**<0.001**	33.262 (2)	**<0.001**	57.776 (2)	**<0.001**	41.058 (2)	**<0.001**

IR, interference resolution; CM, context monitoring; WM, working memory; Δχ^2^, difference in Satorra–Bentler scaled χ^2^ between nested and comparison models; Δdf, difference in degrees of freedom between nested and comparison models. Bolded values p-values represent cases where the more complex model shows significantly better model fit compared to the simpler model.

Finally, the degree of differentiation of these factors from Common EF was unclear; factor correlations for structures in which a 3-factor solution was selected suggest WM differentiates by 5th grade (*M*_*WM and CM*_ = 0.40; *M*_*WM and IR*_ = 0.54), however, a persistent high degree of overlap between CM and IR (*M*_*IR and CM*_ = 0.69) across cohorts leaves open the question of whether one or both of these components would be distinguishable from Common EF (see Supplementary Table 6–10 for full list of factor loadings and correlations). Without statistical methods to determine when components become distinct from both other EFs and Common EF, the use of latent variable models to answer questions about the differentiation hypothesis becomes even more untenable.

#### 3.2.2. Network analysis

Next, we demonstrate how using network analysis to treat EF task performances as an interconnected set of cognitive processes leads to insights into their development, which were not revealed using latent variable modeling. Network analysis provided a data-driven method for grouping task performance according to strength of in-group performance compared to out-group performance, resulting in EF component construction that was not restricted by theoretical assumptions of which tasks draw on each EF component. Further, because we used partial correlations to form networks, the degree of differentiation of components identified with this method is unambiguous; components are only identified if they are distinct from the unifying Common EF component. Thus, network analyses allow for the examination of component grouping after Common EF is accounted for.

**FIGURE 2:**
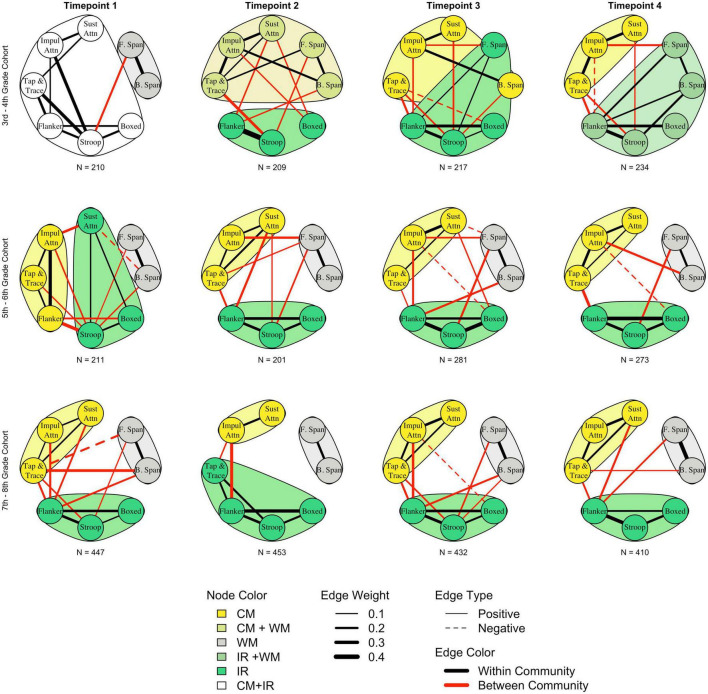
Results of network analysis and community detection for each cohort and timepoint. Strength of the connection between task performance is indicated by line thickness; thicker lines indicate a stronger relationship between two tasks. Edges between -0.1 and 0.1 are not shown for visualization purposes. Connections between tasks are further categorized as either within community (black) or between community (red); weaker and fewer between community connections compared to within community connections is consistent with more distinct communities. Community detection algorithms indicate a two-community organization for the 3rd–4th grade cohort that differentiates into a three community structure by about 5th grade. Fluctuations in grouping and magnitude of edge weights across older cohorts suggests continued subtle development for older students. WM, working memory; CM, context monitoring; IR, interference resolution; B. span, backward spatial span; F. span, forward spatial span; Sust Attn, sustained attention; Impul Attn, impulsive attention.

Concerning the number of components, community detection results (Figure 2) revealed that the EFs examined in this study were organized into two communities through grades 3 and 4, then stabilized into a three-community structure by 5th grade. Yet even through 8th grade, the relationships between tasks continued to evolve over time. Both the CFA and network analytic methods indicated the organization of EF task performances was most variable early in development through grades 3 and 4. However, unlike latent variable modeling, network analysis showed that while the *number* of communities for the 3rd–4th grade cohort was consistent across timepoints, the *composition* of these communities was variable. In this youngest cohort, community detection analysis consistently suggested two of the three theorized components combined into a single component, though similar to the 2-factor solutions tested in the CFA, which component was distinct differed across all four timepoints. Network analysis showed WM was distinct at timepoint 1, IR at timepoint 2, both IR and CM at timepoint 3, and CM at timepoint 4. EF organization for the older cohorts, though, was relatively stable. For both the 5th–6th grade and 7th–8th grade cohorts, the tasks almost always formed three communities with groupings consistent with those predicted by theory. However, for the 5th-6th grade cohort, at timepoint 1, Sustained Attention and Flanker switched communities, grouping with IR and CM communities respectively. Further, at timepoint 2, Tap and Trace was grouped with IR tasks for the 7th–8th grade cohort. Thus, while the EFs examined in this study can be organized into at least three distinct components by about 5th grade, network analysis suggests organization of the IR and CM components in particular continue to undergo refinement across the developmental period examined here. See Supplementary material for additional analyses supporting the results of the community detection analysis.

As indicated by the varying line thickness connecting tasks across models in Figure 2, connections between tasks both within and between communities waxed and waned over development, suggesting the organization of these EFs continued to be refined over time. See Supplementary Figure 6 for estimates for all edge weights with parametric bootstrapped 95% confidence intervals. A unique benefit of network analysis is our ability to leverage the resulting network metrics to quantify and compare the degree of network stability across cohorts. Specifically, we can determine how stable a network is by examining how strongly individual network connections correlate across timepoints for a given cohort. For example, while the strength of individual connections between task performances (e.g., Flanker and Stroop) might increase or decrease over time, these changes are occurring in similar ways over time for a given cohort, the network would be considered more stable in that the organization of task performance is unlikely to change. We used a one-way ANOVA to directly interrogate whether correlations between network connections (Table 4) were more variable in younger cohorts compared to older cohorts. Results revealed these correlations indeed significantly differed across cohorts (*F*(2,15) = 11.29, *p* = 0.001, η^2^ = 0.60). Tukey post-hoc tests showed correlations between the 3rd–4th grade cohort networks connections were significantly lower than correlations between both the 5th–6th grade cohort networks (*M*_*difference*_ = 0.34, 95% CI [0.09–0.58], *p* = 0.008) and the 7th–8th grade cohort networks *(M_*difference*_* = 0.43, 95% CI [0.18, 0.68], *p* = 0.001). Correlations between network connections over time did not differ significantly between these two older cohorts though (*M_*difference*_* = 0.09, 95% CI [-0.15, 0.35], *p* = 0.60). Thus, the period between 3rd and 4th grade is further supported as one in which the organization of EFs is undergoing larger degrees of change compared to the period between 5th and 8th grade, which may show more incremental change. Together, the results of the community detection analysis and the between-cohort differences in network connection correlations illustrate how a holistic examination of the EF system that accounts for Common EF can reveal novel insights into how these processes develop, beginning to resolve the inconsistencies across the literature that have emerged from the use of a reductionist framework that treats components as distinct, but correlated constructs.

**TABLE 4:** Correlations of network connections between network models.

	Timepoint	3rd–4th grade cohort				5th–6th grade cohort				7th–8th grade cohort			
		1	2	3	4	1	2	3	4	1	2	3	4
3rd–4th grade cohort	1	1.00											
	2	0.40 [0.03, 0.67]	1.00										
	3	0.36 [-0.02, 0.64]	0.32 [-0.06, 0.62]	1.00									
	4	0.41 [0.05, 0.68]	0.32 [-0.06, 0.62]	0.45 [0.09, 0.7]	1.00								
5th–6th grade cohort	1	0.48 [0.13, 0.72]	0.56 [0.23, 0.77]	0.54 [0.2, 0.76]	0.43 [0.07, 0.69]	1.00							
	2	0.37 [0, 0.65]	0.34 [-0.04, 0.63]	0.41 [0.04, 0.68]	0.51 [0.17, 0.74]	0.39 [0.01, 0.66]	1.00						
	3	0.43 [0.07, 0.69]	0.48 [0.13, 0.72]	0.47 [0.12, 0.72]	0.43 [0.07, 0.69]	0.75 [0.52, 0.88]	0.67 [0.4, 0.84]	1.00					
	4	0.36 [-0.01, 0.65]	0.51 [0.18, 0.74]	0.64 [0.34, 0.82]	0.55 [0.22, 0.76]	0.62 [0.32, 0.81]	0.58 [0.27, 0.79]	0.63 [0.33, 0.81]	1.00				
7th–8th grade cohort	1	0.36 [-0.02, 0.65]	0.55 [0.23, 0.77]	0.42 [0.05, 0.68]	0.43 [0.07, 0.69]	0.57 [0.25, 0.78]	0.41 [0.04, 0.68]	0.60 [0.3, 0.8]	0.52 [0.18, 0.75]	1.00			
	2	0.34 [-0.04, 0.63]	0.54 [0.21, 0.76]	0.50 [0.16, 0.74]	0.42 [0.05, 0.68]	0.71 [0.46, 0.86]	0.60 [0.29, 0.79]	0.72 [0.47, 0.86]	0.69 [0.42, 0.84]	0.66 [0.38, 0.83]	1.00		
	3	0.54 [0.21, 0.76]	0.62 [0.33, 0.81]	0.39 [0.02, 0.67]	0.47 [0.12, 0.72]	0.72 [0.47, 0.86]	0.65 [0.37, 0.82]	0.78 [0.58, 0.9]	0.69 [0.42, 0.84]	0.61 [0.31, 0.8]	0.85 [0.69, 0.93]	1.00	
	4	0.55 [0.22, 0.77]	0.63 [0.34, 0.81]	0.36 [-0.02, 0.65]	0.51 [0.17, 0.74]	0.55 [0.22, 0.76]	0.63 [0.33, 0.81]	0.54 [0.21, 0.76]	0.62 [0.33, 0.81]	0.61 [0.3, 0.8]	0.64 [0.35, 0.82]	0.64 [0.35, 0.82]	1.00

## 4. Discussion

This study exemplified a feasible analytical technique for testing the differentiation hypothesis and for revealing new insights into the developmental trajectories of EFs. It further demonstrated how methodological choices can influence conclusions and interpretations around the organization of EFs, particularly in developmental populations. By comparing and contrasting the results across analytic techniques, we can bring a new lens to the inconsistencies in the number of EF components in children reported in the literature to date and, with further investigation, resolve them. Ultimately, this work can lay the groundwork towards building a clearer consensus on which EFs emerge on what timeline, and what factors might influence their development.

By applying network analysis techniques, we established a clear developmental timeline of EF organization in our sample and revealed several critical insights into how three EFs examined in the current study evolve over time. First, while both modeling methods used in our analyses point to organization of the examined EFs stabilizing around 5th grade, network analyses were unambiguous in the number of EF components at each timepoint. Network analyses of this sample revealed that a single, undifferentiated component of EF is an unlikely organization for any age in grades 3 through 8. Second, both methods suggest greater variability in the 3rd–4th grade cohort and continued refinement from 5th through at least 8th grade, but only network analysis revealed which EFs are developing and in what way. Our methods revealed that the variability in the 3rd– 4th grade cohort sample was likely due to development and not to traditional constraints such as sample size and measurement differences. Finally, unlike latent variable analysis, the metrics generated from network analyses were used to gain further insight into the development of EFs and develop new hypotheses around their trajectories.

This study presents innovative methods for understanding precisely how EFs differentiate across middle childhood. Adaptive algorithms in our EF assessments allow us to meet the learner where they are, regardless of ability and without making assumptions about skill level according to demographic variables such as age and allow for multiple assessments within-subject over time. Using our novel technology, we administered assessments to large groups of children at once, affording us a larger sample size for each age group studied. These large samples of students, who completed the same tasks that presented a similar degree of challenge according to individual performance, represent a unique dataset from which to understand three EFs. Paired with simulation results, we can be more confident that differences seen between cohorts are developmentally-related—not merely due to differences in sample size or task difficulty. In this way, we overcame one pernicious limitation in the extant literature, which has commonly had to use different tasks for different age ranges (e.g., McAuley and White, 2011; Camerota et al., 2020; though see Van der Ven et al., 2012; Boelema et al., 2014) or seen ceiling effects in performance by older students (e.g., Lee et al., 2013).

Using a network analytic approach and leveraging the power of this dataset, we were able to explore new avenues for understanding the development of EF as a dynamic interconnected network of skills that can align behavioral and neural models. Our series of analyses provide converging evidence that the period from third to fourth grade is one of great change in the structure and organization of EFs compared to later periods in development. Not only did both latent variable and network analysis show a greater degree of variability in the model that best represents organization of EFs, but the between network correlations between edge weights support characterizing organizations as “unstable”. This pattern of findings may suggest individual differences in component differentiation that should be explored in future research. Experience may drive differentiation rates to differ across children. Further, individual differences in differentiation rates may also explain differences in the number of EF components found in this age range. For example, studies have shown students might differentially employ EFs based on, for example, pubertal stage, socioeconomic background, et cetera (Haft and Hoeft, 2017; Doebel, 2020). Such differences in the way individuals employ EFs may also impact the trajectory of differentiation of these EFs. Understanding the potential paths in development and how they can be influenced by life experience will be critical in fostering continued growth of EF skills (Best and Miller, 2010).

In the current study, network analysis allowed us to go beyond assessing the stability of the number of components across development and extend our assessment to the stability of component *composition*. As discussed, the 3rd–4th grade cohort in this study was highly variable across time, showing a different combination of components at each of the four timepoints examined. However, this variation in organization was not restricted to the youngest cohort; the 5th–6th grade cohort studied here showed a non-hypothesized organization at timepoint 1, namely, Flanker grouping with context monitoring rather than interference resolution, and Sustained Attention grouping with interference resolution. This finding, consistent with prior work showing protracted development of EF skill (Davidson et al., 2006), emphasizes that EFs may manifest or be deployed differently across development, and tasks shown to measure one construct in adults may measure a different one in children (Morra et al., 2018). Such potential differences in how EFs might be employed to accomplish a task across development were missed when using a latent variable model approach, and may help explain the inconsistencies in the extant literature regarding the number of components in this age range (Lee et al., 2013). Latent variable analysis does not allow for statistically comparing models with different configurations of the same indicators. As such, alternate configurations are often not investigated. During a period of such developmental instability, the differences in the tasks used to measure each component and the metric of skill on each of those tasks across studies could result in many acceptable models of the data. Without a data-driven method for determining which EF component a task reflects, researchers are left with an untenable number of configurations to test. Indeed in the current study, such configurational differences were missed with factor analysis, since the theory-driven configuration of EFs fit reasonably well, and there was no indication a different configuration might better represent EF constructs. Considering alternative approaches such as the network analysis shown here can add to our understanding of the measurement approach that best represents EFs across the lifespan (Camerota et al., 2020).

Importantly, the use of network analysis to test the differentiation hypothesis allowed for the examination of how different EFs become distinct from not only each other, but from Common EF. To date, only one other investigation to our knowledge has used analytic methods that support such an investigation (Hartung et al., 2020). While this investigation examined different EFs than those studied here (specifically, Working Memory, Switching, Updating, and Inhibition), the results are largely complementary. Specifically, Hartung and colleagues analyses indicated that in younger children age 8–10, EFs were highly correlated with one another, suggesting little differentiation between Common EFs and individual components at this age. Further, Inhibition, most similar to the CM and IR examined here, became increasingly differentiated by about age 10, consistent with the finding from the current study that organization of CM, IR, and WM stabilized around 5th grade, or age 10. Finally, a primary finding from Hartung et al.’s (2020) investigation was the lack of a uniform pattern of development across either components or individual tasks, suggesting a more nuanced pattern of developmental trajectories, consistent with the findings from the current study. Both studies underscore the importance of carefully considering which components are measured in what way, and whether the relationships between tasks and EFs seen in adults holds true for childhood populations.

### 4.1. Limitations and future directions

This study makes significant strides in our approach to measure and model EFs, improving on several critical limitations in the field. Yet, further advancements are needed to build upon and address limitations of this work, particularly regarding the scope of EFs assessed and the availability of statistical methods to compare network models longitudinally.

#### 4.1.1. EF measurement

Developing a novel, adaptive battery of EF tasks for all ages and abilities was not without its challenges, and a future iteration of this battery that addresses many of the challenges encountered here is already underway. This iteration, called ACE Explorer (ACE-X) is currently undergoing large-scale norming and validation with a nationally representative sample across ages 7–107. A key challenge with using ACE-C concerned the design decisions made when modifying tasks for large-group assessment and to incorporate adaptive algorithms. Specifically, in this study, the WM component was only indexed by two measures, which limited the type of latent variable model that could be constructed and tested here. While a third task hypothesized to measure WM, Filter, was originally included in the ACE-C battery, it used a different adaptive mechanism, which resulted in age-related differences in challenge level, and ultimately its exclusion from the current analysis. Consequently, we could not test certain factor configurations without rendering the models uninformative. In ACE-X, we have aligned the adaptive mechanism to use the response window in the same manner as the majority of other tasks in the battery, which has resulted in more consistent challenge-levels across age groups.

Further, as with any investigation that does include an exhaustive assessment of all potential EFs, the conclusions concerning developmental trajectory of EFs can only be applied to what was examined. The components examined via ACE-C were not intended to be an exhaustive list of potential EF components, and notably, not all components intended to be measured with ACE-C were able to be included. Due to time constraints associated with in-school testing sessions, we were limited in the number of tasks that could be administered, and we chose to focus on tasks commonly used to assess EFs and cognitive control across both the adult and developmental literature to better bridge our understanding of these constructs across the lifespan. Further, while we did administer a task intended to assess the cognitive flexibility component of EF, a technical malfunction in the analytics for this task prevented its inclusion in the current study. As such, one prominent EF component was not assessed here, though this issue has been corrected in the ACE-X battery.

Additionally, careful consideration must be given to the terms that are used to discuss EF components, and how those terms are reflected by the task designs used in each investigation. For example, the Stroop task has been considered to measure inhibition when the verbal response mechanism is used, but interference resolution when a motor response mechanism is used, as is the case in the current study. Further, while the components put forth by Miyake and colleagues (Miyake et al., 2000b; “updating”, “inhibitory control”, and “cognitive flexibility”) are the most frequently examined components (Karr et al., 2018) they are often inconsistently defined across the literature. In particular, the “inhibitory control” component is often measured with a combination of tasks that involve both the “interference resolution” and “context monitoring” aspects of cognitive control (see Diamond, 2013 for review). However, neural data from both children and adults indicate these are indeed two separate components (Bunge et al., 2002). By including additional tasks (e.g., Boxed and Tap and Trace) and separating inhibitory control-related tasks into those in which a response must always be made (interference resolution) and those in which a participant must decide whether to make a response or not (context monitoring), the ACE-C battery is able to bring further specificity to the characterization of EFs in middle childhood.

Similarly, though, the “working memory” component of EF would benefit from increased precision around its definition, and therefore measurement. The field has not yet reached a consensus on whether “short term memory” is distinct from “working memory” and whether these constructs might differ across development as this component evolves. While the inclusion of both a forward and backward span in the ACE-C battery was done in keeping with their widespread use in clinical practice to assess what is referred to as “working memory” (see Berch et al., 1998), these two tasks do not exhaustively capture all potential aspects of the construct. Indeed, in this investigation we used the term “working memory” rather than “updating” as is used in the most commonly cited model of EF components (Miyake et al., 2000b) because the Forward Spatial Span task does not strictly fit with the component conceptualized as “updating”. By including additional tasks that tap different aspects of “working memory”, such as the Filter task that examines the ability to remember task-relevant information while ignoring task irrelevant information (Luck and Vogel, 1997), we can further understand the composition of this construct and bring increased specificity to how it is discussed and measured.

Finally, future directions for the ACE battery include increasing its capabilities as a measurement tool of multiple components of EF. First, ACE-X has been made more inclusive by using a color palette compliant with the Americans with Disabilities Act to ensure individuals who are colorblind can use the battery. Second, to build on to the engaging design that afforded us high retention and compliance rates in this study, ACE-X incorporates the battery of tasks into a cohesive story to further motivate participants to complete the full battery. Finally, the large-scale validation efforts and norming with a nationally representative population will further allow us to replicate the results shown here in additional populations, including within sub-populations represented but not separately examined in this study (e.g., students with learning disabilities). In this way, we will be able to replicate and extend the results of the current study, to better understand additional factors that may impact the developmental trajectory described here.

#### 4.1.2. EF modeling

This study demonstrated a new approach to modeling EFs that better accounts for the unity while examining the diversity of EFs. Yet, this methodological approach must continue to be built upon to fully model the development of EFs. Indeed, there were methodological challenges related to comparing two analytical approaches in testing the differentiation hypothesis. For example, we intentionally did not explicitly model the dependency of multiple observations per student that occurs with longitudinal data in either analytic approach. While it is possible to model using factor analysis, development of network models that can handle longitudinal data are still in their infancy (though see Deserno et al., 2021). To keep the general modeling strategy consistent and inferences comparable, we treated all observations as independent in both approaches. However, this strategy is unlikely to have affected the results for two reasons. First, without accounting for within-person changes, within-cohort comparisons were more conservative than necessary. Second, we did not perform tests that were likely to be affected by treating observations as independent. Nonetheless, as network analytic methodology continues to advance, so too must the methods used to reveal the evolution of EF structure advance.

Further, neither modeling approach was able to simultaneously account for Common EF *and* provide statistical comparisons between models of differing complexity. With latent variable analysis, it is a straightforward process to compare whether a model with more factors fits statistically better than a model with fewer factors. These capabilities, though, are currently limited with network models (though see Epskamp et al., 2021). Community detection algorithms provide a likely grouping for task performances, but there is no index to statistically determine whether a two-community network explains EFs just as well as a three-community network, for example. However, existing methods for accounting for Common EF in the latent variable approach preclude such statistical comparisons between models, leaving the theoretical problem of how to account for Common EF in the context of differentiation of components with this approach unresolved. To date, the benefits of the network analysis approach, which accounts for commonality among all EF task performances rather than treating it as a separate component entirely, presents a promising solution for accounting for Common EF. The rapidly emerging statistical approaches for testing network model complexity position this technique as the path forward in establishing the developmental trajectory of EFs.

The future potential for network analysis to help us understand complex cognitive constructs is bright. Researchers in related fields have already begun to capitalize on information gained from taking a network analytic perspective to understand other cognitive processes. For example, Kan et al. (2020) demonstrated how fit statistics can be obtained for network models, allowing a direct comparison between network and latent variable models. As such, future research could directly compare a variety of configurations of EF modeled using latent variable analysis to those using network analysis to determine which organization best fits observed EF performance. While outside the scope of the current paper, researchers in the field of intelligence have used this approach to show that modeling aspects of intelligence as being mutually and reciprocally related through a network framework is favored over modeling an overarching umbrella component (“g”) in a latent variable framework (Kan et al., 2019). Given this field’s similar dilemma around how to quantify developmental differentiation in the presence of task commonality (Molenaar et al., 2010), we anticipate such investigations in EFs will be similarly fruitful for determining which modeling approach better reflects the unity and diversity of EFs and for elucidating the mechanisms through which skill changes arise.

Further, as methods for appropriately modeling longitudinal data emerge, network analysis provides an avenue for understanding the potential reciprocal relationships among EFs over time (Deserno et al., 2021). For example, in a separate study we are examining how growth in performance on individual tasks are connected. By using a network framework for investigating EF skill growth, we can evaluate whether the same communities formed when modeling contemporaneous ties between task performances also emerge when looking at their patterns of growth across time. Such evidence would reinforce the identity of the communities as distinct components of EF and allow us to answer whether components of EF emerge independently or in tandem with other components.

Such insights into the development of EFs are critical for advancing our understanding of how they influence, and can be influenced by, internal and external factors. For example, EFs are often the focus of educational interventions with the goal of improving academic-related outcomes (see e.g., Diamond and Lee, 2011; Titz and Karbach, 2014; Jacob and Parkinson, 2015). Network analysis is well-poised to generate hypotheses regarding which EF tasks or components might be more likely to transfer outside a training regime, which can then guide future training studies. Indeed, the findings from the current study provide a clear set of testable hypotheses: given that the cross-sectional network models found here suggest that WM is less strongly connected to other EF components, future training studies should test the hypothesis that training a highly connected component such as IR would be more likely to result in transfer to other EFs compared to training on the less-well connected WM component.

### 4.2. Conclusion

The findings from this study showcase how advances in assessing EFs and an increasingly popular modeling technique, network analysis, can be applied to the field of EFs to better align behavioral and neural investigations. The dual paradigm shifts to network analysis using adaptive measures provide a promising pathway for refining and specifying our understanding of how EFs develop. These insights can in turn be applied to advance our understanding of EFs’ wide-reaching impact on factors related to physical and cognitive health across the lifespan (Zelazo et al., 2016). Together, our improved methodological approaches to *measuring* EFs can lead to the development of improved methods for *supporting* EFs and providing students the proper foundation they need for learning and future educational success.

## Data availability statement

The original contributions presented in this study are publicly available. This data can be found here: https://osf.io/scpkm/.

## Ethics statement

The studies involving human participants were reviewed and approved by The University of California San Francisco Institutional Review Board. Written informed consent to participate in this study was provided by the participants’ legal guardian/next of kin.

## Author contributions

JA, SB, FH, BM, JM, MR-L, AG, and MU conceived of and designed the study. JY and MU collected the data. JY, KO’L, EF, and MU analyzed the data. JY, KO’L, and MU wrote the manuscript. All authors discussed the results and contributed to editing the manuscript.
