# Supplementary material for: Better together: novel methods for measuring and modeling development of executive function diversity while accounting for unity
Source: Front Hum Neurosci. 2023 Jul 24;17:1195013. doi: 10.3389/fnhum.2023.1195013 (PMC10405287; doi:10.3389/fnhum.2023.1195013)
Supplement: Supplementary file 1 [file Data_Sheet_1.docx]

Supplementary Material

**Better together: Novel methods for measuring and modeling development of executive function diversity while accounting for unity**

**Jessica Wise Younger^1*^, Kristine D. O’Laughlin^1^, Joaquin A. Anguera^1^, Silvia A. Bunge^2^, Emilio E. Ferrer^3^, Fumiko Hoeft^4,5^, Bruce D. McCandliss^6^, Jyoti Mishra^7,8^, Miriam Rosenberg-Lee^9^, Adam Gazzaley^1,10^, and Melina R. Uncapher^1,11*^**

***Corresponding Authors**
Jessica Wise Younger: [jessica@youngers.org](mailto:jessica@youngers.org)
Melina R. Uncapher: [muncapher@aerdf.org](mailto:muncapher@aerdf.org)

# 1 Supplemental Text

The community detection results reflected a clear pattern of emerging differentiation of the EFs studied here with a single undifferentiated component being unlikely for any cohort at any timepoint. To confirm this result, we performed the same community detection techniques on a set of simulated data in which the true-generating model was a one-factor CFA. These results showed that in a one-factor model, the most frequently selected community structure was selected in only 1.8% of 1,000 iterations. By comparison, the selected community structures in the observed data were the solution in between 45% and 96% of the iterations.

We additionally took further steps to ensure the apparent lack of stability in EF structure for the 3rd-4th grade cohort suggested by the results of community detection analyses were not due to differences in sample sizes across cohorts and timepoints. We performed resampling with replacement for each network 500 times with 1,000 iterations of community detection and sample size fixed at 500 to confirm the consistency of the formed communities. In all cases except for the 3rd-4th grade cohort at timepoint 3, the same organization was selected most frequently across iterations in the resampled datasets and the observed data. At timepoint 3 for the 3rd-4th grade cohort, resampling results suggested Boxed and Tap and Trace changed which communities they were grouped with compared to the model selected with the observed data. However, this model, while the most frequently selected model in the resampled data, had the lowest selection rate of all models: 12.2% of the 500 resampled datasets. Together, the resampling results suggest the instability of model selection in the 3rd-4th grade cohort is likely due to developmental differences, rather than sample size or measurement differences compared to the older cohorts. A comparison of most frequent solutions between the observed data and resampled data across all timepoints and cohorts is presented in Table S11.

# 2 Supplementary Figures and Tables

*
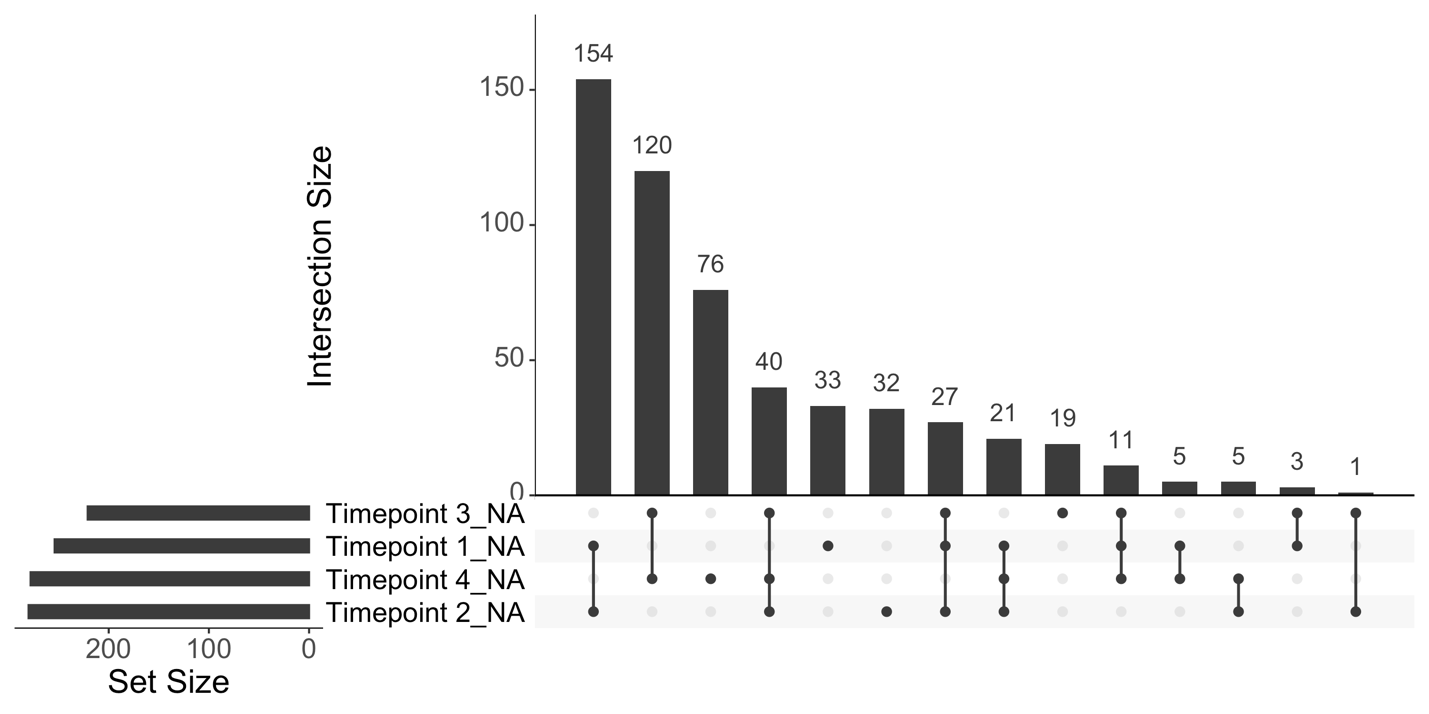
***Figure S1.** Pattern of missing data across timepoints. Not depicted are 716 cases that contributed data at all four timepoints.

**Figure S2.** Example of stimuli and task schematics for each ACE-C task. All tasks had a trial result interval of 200ms and an inter trial interval of 1000ms following the target or probe. The trial result interval was 500ms for the Forward and Backward Spatial Span tasks.


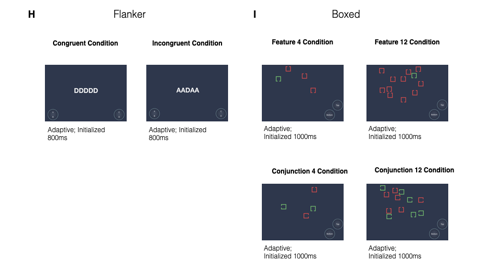


*
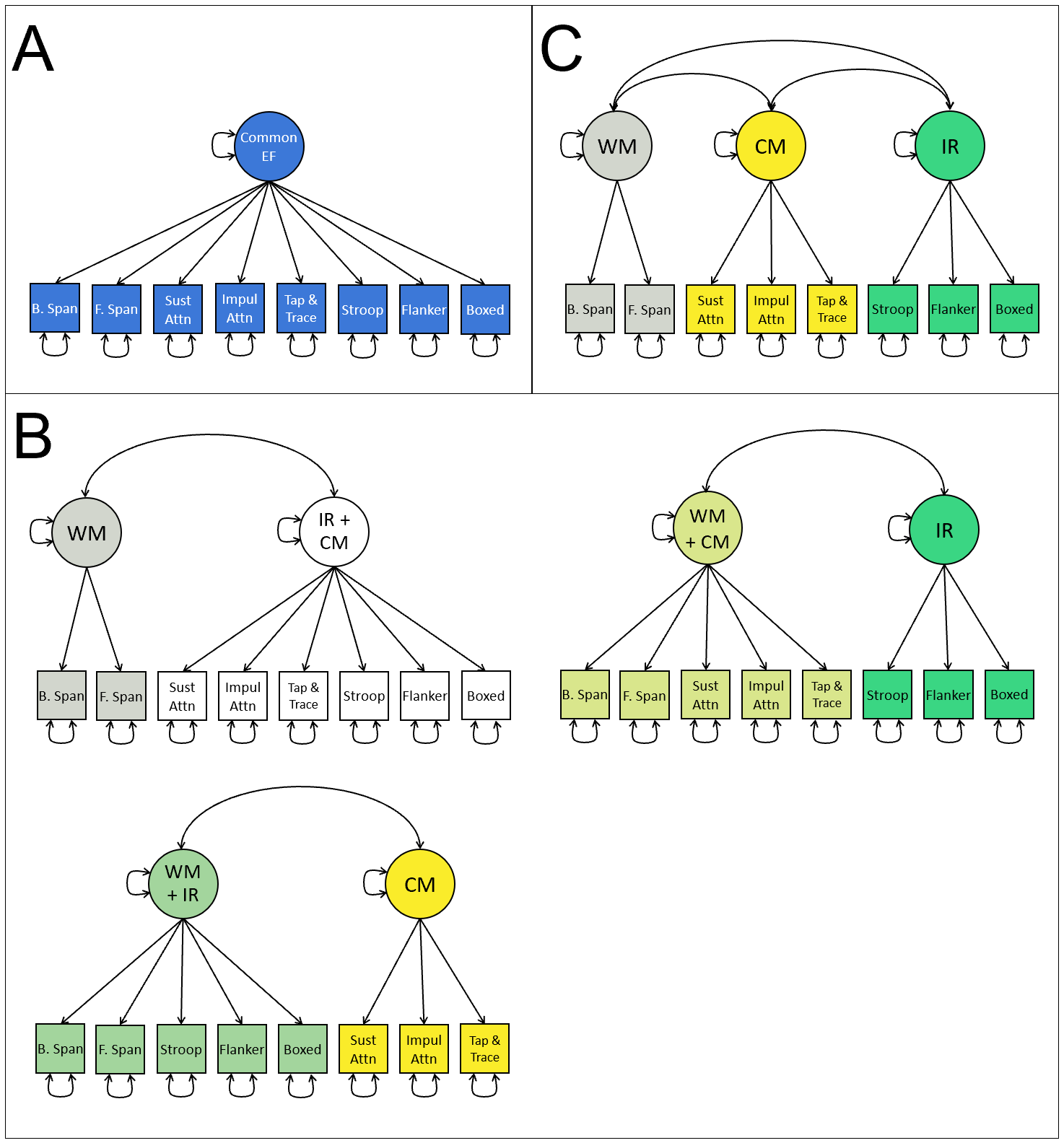
***Figure S3.** Potential organization of executive function factor structure. EF = executive function; WM = Working Memory; IR = Interference Resolution; CM = Context Monitoring; B. Span = Backward Spatial Span; F. Span = Forward Spatial Span; Sust Attn = Sustained Attention; Impul Attn = Impulsive Attention. Panel A shows a 1-factor model where all tasks load onto a single factor of common executive function; panel B shows three possible combinations of factors to form a 2-factor model; and panel C shows the 3-factor model of executive function.


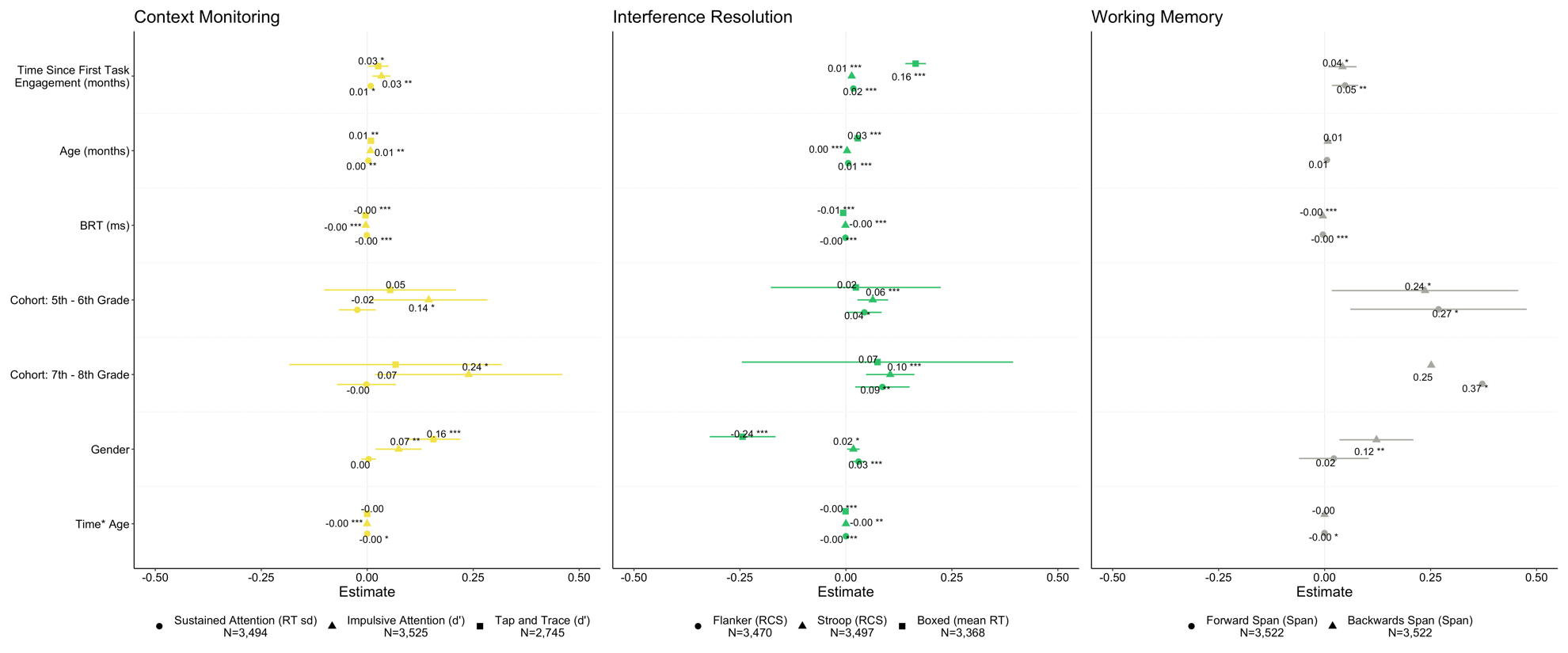


**Figure S4.** Estimates for each fixed effect in linear mixed models of task performance. Lines represent 95% confidence interval.

****p* < 0.001, ***p* < 0.01, **p* < 0.05*; RCS = rate correct score; RT = reaction time, SD = standard deviation. Boxed and Sustained Attention performance scores have been transformed so that positive estimates indicate higher performance for all tasks and estimates are on similar scale across tasks.

**
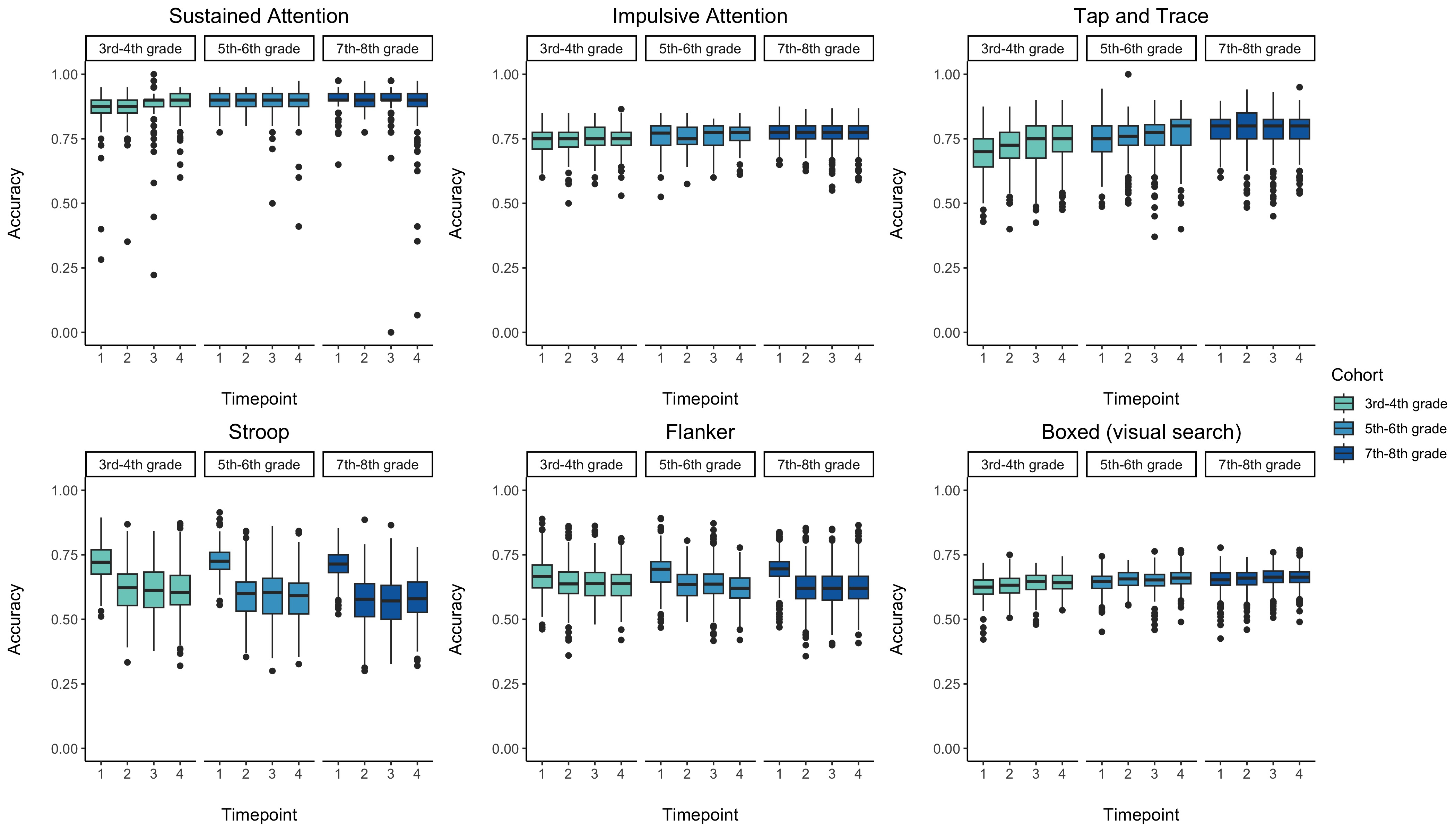
**

**Figure S5.** Boxplots of in-game accuracy for tasks with an adaptive response window

**Figure S6.** Bootstrapped 95% Confidence Intervals of estimated edge-weights for each network of EF task performance. The red line indicates the sample values and the surrounding gray area the bootstrapped CIs. Each horizontal line represents one edge of the network. B. Span = Backward Spatial Span; F. Span = Forward Spatial Span; SUST ATTN = Sustained Attention; IMPUL ATTN = Impulsive Attention.

|  | ***N Datasets Before Cleaning*** | ***N Too Few Trials*** | ***N Below Chance Performance*** | ***N Outlier Task Performance*** | ***N Influential Observation in Linear Model*** | ***Total Datasets Removed*** |
| --- | --- | --- | --- | --- | --- | --- |
| ***Forward Spatial Span*** | 3951 | 0 (0%) | 0 (0%) | 0 (0%) | 1 (0.03%) | 1 (0.03%) |
| ***Backward Spatial Span*** | 3949 | 0 (0%) | 0 (0%) | 0 (0%) | 1 (0.03%) | 1 (0.03%) |
| ***Sustained Attention*** | 4003 | 0 (0%) | 16 (0.40%) | 68 (1.70%) | 1 (0.02%) | 85 (2.12%) |
| ***Impulsive Attention*** | 4008 | 0 (0%) | 17 (0.42%) | 36 (0.09%) | 1 (0.02%) | 54 (1.35%) |
| ***Tap & Trace*** | 3104 | 5 (0.16%) | 21 (0.39%) | 26 (0.84%) | 1 (0.03%) | 44 (1.42%) |
| ***Stroop*** | 3979 | 4 (0.10%) | 5 (0.13%) | 54 (1.36%) | 16 (0.40%) | 79 (1.99%) |
| ***Flanker*** | 3972 | 3 (0.08%) | 28 (0.70%) | 49 (1.23%) | 8 (0.20%) | 88 (2.22%) |
| ***Boxed*** | 3986 | 15 (0.38%) | 123 (3.09%) | 68 (1.71%) | 21 (0.53%) | 227 (5.69%) |

Table S1*. Count of Task Datasets Removed During Cleaning Procedures*

*Note*: Datasets were evaluated and removed on a task-level basis, not at the participant level.

Table S2. *Correlations, Means, and Standard Deviations of Performance on Executive Function Tasks with Basic Response Time Regressed for 3^rd^-4^th^ Grade Cohort*


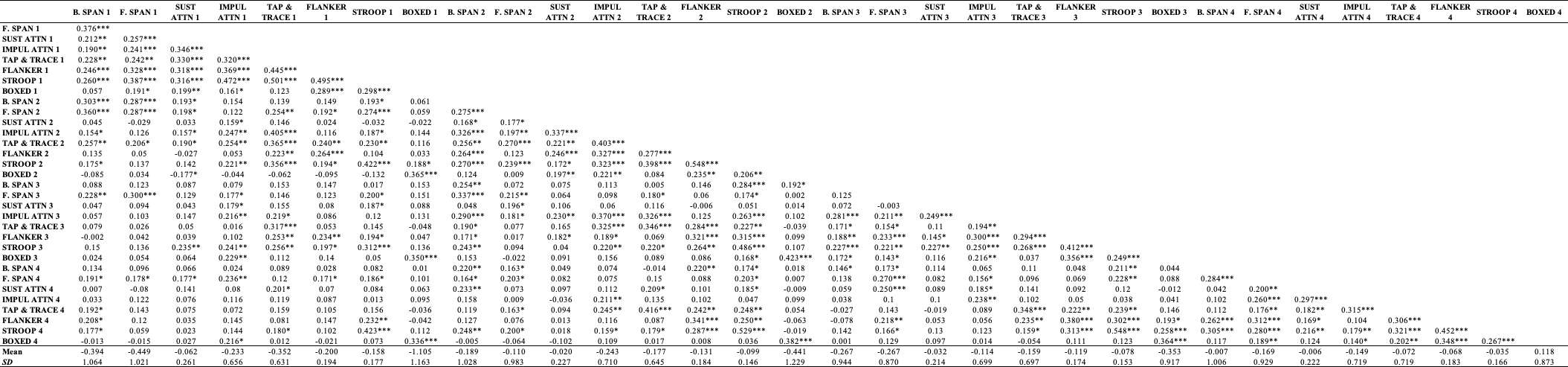


*Note.* **p* < 0.05; ***p* < 0.01; ****p* < 0.001. B. Span *t* = Backward Spatial Span at time *t*; F. Span *t* = Forward Spatial Span at time *t*; Sust Attn *t* = Sustained Attention at time *t*; Impul Attn *t* = Impulsive Attention at time *t*; Tap & Trace *t* = Tap and Trace at time *t*; Flanker *t* = Flanker at time *t*; Stroop *t* = Stroop at time *t*; Boxed *t* = Boxed at time *t*.

Table S3. *Correlations, Means, and Standard Deviations of Performance on Executive Function Tasks with Basic Response Time Regressed for 5^th^-6^th^ Grade Cohort*


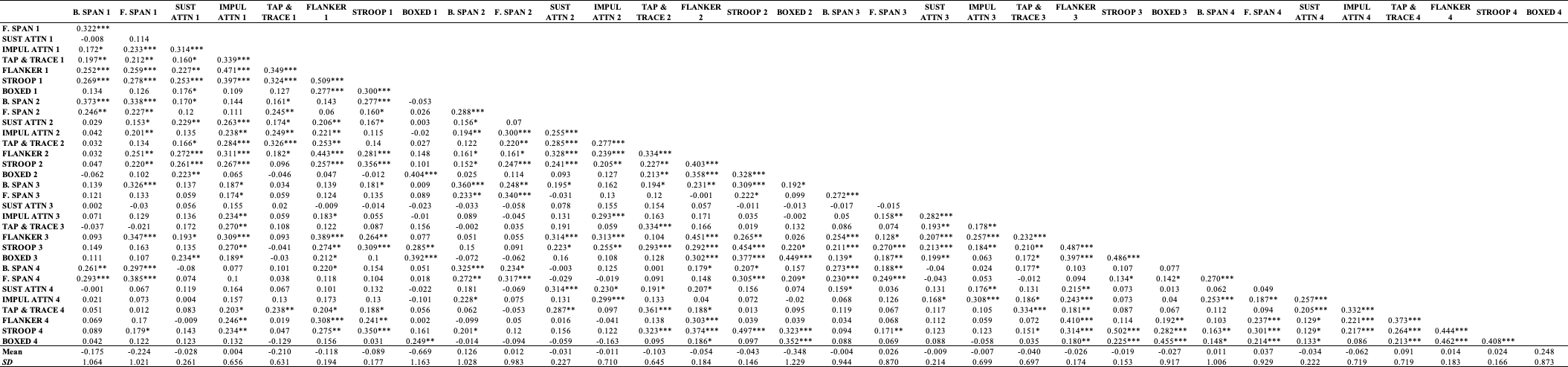


*Note.* **p* < 0.05; ***p* < 0.01; ****p* < 0.001. B. Span *t* = Backward Spatial Span at time *t*; F. Span *t* = Forward Spatial Span at time *t*; Sust Attn *t* = Sustained Attention at time *t*; Impul Attn *t* = Impulsive Attention at time *t*; Tap & Trace *t* = Tap and Trace at time *t*; Flanker *t* = Flanker at time *t*; Stroop *t* = Stroop at time *t*; Boxed *t* = Boxed at time *t*.

Table S4. *Correlations, Means, and Standard Deviations of Performance on Executive Function Tasks with Basic Response Time Regressed for 7^th^-8^th^ Grade Cohort*


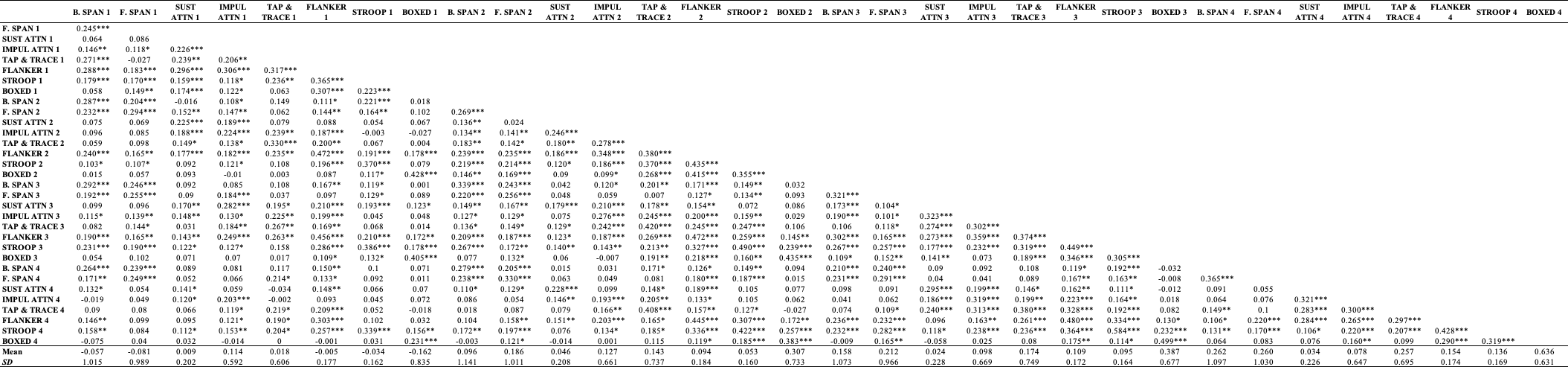


*Note.* **p* < 0.05; ***p* < 0.01; ****p* < 0.001. B. Span *t* = Backward Spatial Span at time *t*; F. Span *t* = Forward Spatial Span at time *t*; Sust Attn *t* = Sustained Attention at time *t*; Impul Attn *t* = Impulsive Attention at time *t*; Tap & Trace *t* = Tap and Trace at time *t*; Flanker *t* = Flanker at time *t*; Stroop *t* = Stroop at time *t*; Boxed *t* = Boxed at time *t*.

Table S5. *Fit Indices for 1-, 2-, and 3-Factor Models of Executive Function*

| **Cohort** | **Timepoint** | **Model** | $\boldsymbol{\chi}^{\boldsymbol{2}}$ | ***df*** | ***p*** | **RMSEA (90% CI)** | **CFI** | **AIC** | **BICc** |
| --- | --- | --- | --- | --- | --- | --- | --- | --- | --- |
| *3^rd^-4^th^ grade cohort* | 1 | 1-Factor | 28.942 | 20 | 0.089 | 0.046 (0.000, 0.081) | 0.972 | 2091.328 | 2095.613 |
|  |  | 2-Factor (IR with CM)^a,b,c,d^ | 16.535 | 19 | 0.621 | 0.000 (0.000, 0.052) | 1.000 | 2081.227 | 2085.690 |
|  |  | 2-Factor (WM with CM) | 28.74 | 19 | 0.070 | 0.049 (0.000, 0.084) | 0.969 | 2093.183 | 2097.646 |
|  |  | 2-Factor (WM with IR) | 28.85 | 19 | 0.068 | 0.050 (0.000, 0.084) | 0.969 | 2093.091 | 2097.554 |
|  |  | 3-Factor^a,b^ | 16.417 | 17 | 0.495 | 0.000 (0.000, 0.061) | 1.000 | 2084.938 | 2089.759 |
|  | 2 | 1-Factor | 42.713 | 20 | 0.002 | 0.074 (0.043, 0.104) | 0.899 | 2023.063 | 2027.235 |
|  |  | 2-Factor (IR with CM) | 38.051 | 19 | 0.006 | 0.069 (0.036, 0.101) | 0.916 | 2020.398 | 2024.744 |
|  |  | 2-Factor (WM with CM)^c,d^ | 22.254 | 19 | 0.272 | 0.029 (0.000, 0.070) | 0.986 | 2005.357 | 2009.702 |
|  |  | 2-Factor (WM with IR) | 32.105 | 19 | 0.030 | 0.057 (0.018, 0.091) | 0.942 | 2015.517 | 2019.862 |
|  |  | 3-Factor^a,b^ | 18.713 | 17 | 0.345 | 0.022 (0.000, 0.068) | 0.992 | 2005.74 | 2010.433 |
|  | 3 | 1-Factor | 24.719 | 20 | 0.212 | 0.033 (0.000, 0.070) | 0.972 | 2020.522 | 2025.587 |
|  |  | 2-Factor (IR with CM) | 24.870 | 19 | 0.165 | 0.038 (0.000, 0.075) | 0.965 | 2022.520 | 2027.796 |
|  |  | 2-Factor (WM with CM)^a,b,c,d^ | 21.387 | 19 | 0.316 | 0.024 (0.000, 0.066) | 0.986 | 2019.132 | 2024.407 |
|  |  | 2-Factor (WM with IR) | 23.275 | 19 | 0.225 | 0.032 (0.000, 0.071) | 0.974 | 2021.172 | 2026.447 |
|  |  | 3-Factor | 21.156 | 17 | 0.219 | 0.034 (0.000, 0.074) | 0.975 | 2022.887 | 2028.585 |
|  | 4 | 1-Factor | 46.288 | 20 | 0.001 | 0.075 (0.047, 0.103) | 0.885 | 2340.434 | 2347.292 |
|  |  | 2-Factor (IR with CM) | 43.359 | 19 | 0.001 | 0.074 (0.045, 0.103) | 0.894 | 2339.041 | 2346.185 |
|  |  | 2-Factor (WM with CM) | 42.241 | 19 | 0.002 | 0.072 (0.043, 0.102) | 0.898 | 2337.58 | 2344.724 |
|  |  | 2-Factor (WM with IR)^a,b,c,d^ | 31.717 | 19 | 0.034 | 0.053 (0.015, 0.085) | 0.944 | 2327.581 | 2334.726 |
|  |  | 3-Factor ^a^ | 29.736 | 17 | 0.028 | 0.057 (0.018, 0.090) | 0.944 | 2328.42 | 2336.136 |
| *5^th^-6^th^ grade cohort* | 1 | 1-Factor | 29.285 | 20 | 0.082 | 0.047 (0.000, 0.081) | 0.962 | 1971.696 | 1976.094 |
|  |  | 2-Factor (IR with CM)^c,d^ | 19.434 | 19 | 0.429 | 0.010 (0.000, 0.061) | 0.998 | 1963.853 | 1968.434 |
|  |  | 2-Factor (WM with CM) | 27.789 | 19 | 0.088 | 0.047 (0.000, 0.082) | 0.964 | 1972.386 | 1976.967 |
|  |  | 2-Factor (WM with IR) | 26.008 | 19 | 0.130 | 0.042 (0.000, 0.078) | 0.971 | 1970.28 | 1974.861 |
|  |  | 3-Factor^a,b^ | 16.315 | 17 | 0.502 | 0.000 (0.000, 0.060) | 1.000 | 1964.679 | 1969.627 |
|  | 2 | 1-Factor | 39.38 | 20 | 0.006 | 0.069 (0.036, 0.101) | 0.897 | 1810.579 | 1813.823 |
|  |  | 2-Factor (IR with CM) | 29.893 | 19 | 0.053 | 0.053 (0.000, 0.088) | 0.942 | 1803.739 | 1807.118 |
|  |  | 2-Factor (WM with CM) | 26.581 | 19 | 0.115 | 0.045 (0.000, 0.081) | 0.960 | 1801.019 | 1804.397 |
|  |  | 2-Factor (WM with IR) | 33.282 | 19 | 0.022 | 0.061 (0.023, 0.095) | 0.924 | 1807.597 | 1810.976 |
|  |  | 3-Factor^a,b,c,d^ | 19.092 | 17 | 0.323 | 0.025 (0.000, 0.071) | 0.989 | 1798.024 | 1801.673 |

Table S5. *Continued*

| **Cohort** | **Timepoint** | **Model** | $\boldsymbol{\chi}^{\boldsymbol{2}}$ | ***df*** | ***p*** | **RMSEA (90% CI)** | **CFI** | **AIC** | **BICc** |
| --- | --- | --- | --- | --- | --- | --- | --- | --- | --- |
| *5^th^-6^th^ grade cohort* | 3 | 1-Factor | 56.947 | 20 | < 0.001 | 0.081 (0.057, 0.106) | 0.863 | 2640.671 | 2651.888 |
|  |  | 2-Factor (IR with CM) | 45.769 | 19 | 0.001 | 0.071 (0.045, 0.097) | 0.901 | 2631.344 | 2643.029 |
|  |  | 2-Factor (WM with CM) | 55.165 | 19 | < 0.001 | 0.082 (0.057, 0.108) | 0.866 | 2639.180 | 2650.865 |
|  |  | 2-Factor (WM with IR) | 40.784 | 19 | 0.003 | 0.064 (0.037, 0.091) | 0.919 | 2626.649 | 2638.334 |
|  |  | 3-Factor^a,b,c,d^ | 28.834 | 17 | 0.036 | 0.050 (0.013, 0.080) | 0.956 | 2617.989 | 2630.609 |
|  | 4 | 1-Factor | 65.133 | 20 | < 0.001 | 0.091 (0.067, 0.116) | 0.846 | 2633.798 | 2644.328 |
|  |  | 2-Factor (IR with CM) | 55.520 | 19 | < 0.001 | 0.084 (0.059, 0.110) | 0.875 | 2626.306 | 2637.274 |
|  |  | 2-Factor (WM with CM) | 48.997 | 19 | < 0.001 | 0.076 (0.050, 0.103) | 0.898 | 2619.775 | 2630.742 |
|  |  | 2-Factor (WM with IR) | 42.310 | 19 | 0.002 | 0.067 (0.040, 0.094) | 0.920 | 2614.622 | 2625.590 |
|  |  | 3-Factor^a,b,c,d^ | 34.010 | 17 | 0.008 | 0.061 (0.030, 0.090) | 0.942 | 2608.671 | 2620.517 |
| *7^th^-8^th^ grade cohort* | 1 | 1-Factor | 45.447 | 20 | 0.001 | 0.053 (0.033, 0.074) | 0.917 | 3614.565 | 3636.861 |
|  |  | 2-Factor (IR with CM)^d^ | 33.551 | 19 | 0.021 | 0.041 (0.016, 0.064) | 0.953 | 3604.705 | 3627.929 |
|  |  | 2-Factor (WM with CM) | 43.105 | 19 | 0.001 | 0.053 (0.032, 0.075) | 0.922 | 3614.040 | 3637.264 |
|  |  | 2-Factor (WM with IR) | 39.692 | 19 | 0.004 | 0.049 (0.027, 0.071) | 0.933 | 3611.051 | 3634.276 |
|  |  | 3-Factor ^a,b,c^ | 27.931 | 17 | 0.046 | 0.038 (0.005, 0.062) | 0.965 | 3602.978 | 3628.060 |
|  | 2 | 1-Factor | 54.174 | 20 | < 0.001 | 0.061 (0.042, 0.081) | 0.918 | 3862.815 | 3885.429 |
|  |  | 2-Factor (IR with CM) | 40.496 | 19 | 0.003 | 0.050 (0.028, 0.071) | 0.948 | 3851.435 | 3874.992 |
|  |  | 2-Factor (WM with CM) | 48.840 | 19 | < 0.001 | 0.059 (0.039, 0.080) | 0.928 | 3859.364 | 3882.920 |
|  |  | 2-Factor (WM with IR) | 47.197 | 19 | < 0.001 | 0.057 (0.037, 0.078) | 0.932 | 3857.930 | 3881.486 |
|  |  | 3-Factor^a,b,c,d^ | 33.593 | 17 | 0.010 | 0.046 (0.022, 0.069) | 0.960 | 3848.432 | 3873.872 |
|  | 3 | 1-Factor | 74.930 | 20 | < 0.001 | 0.080 (0.061, 0.099) | 0.873 | 3895.085 | 3916.565 |
|  |  | 2-Factor (IR with CM) | 52.217 | 19 | < 0.001 | 0.064 (0.043, 0.085) | 0.923 | 3874.065 | 3896.440 |
|  |  | 2-Factor (WM with CM) | 66.528 | 19 | < 0.001 | 0.076 (0.057, 0.096) | 0.890 | 3887.100 | 3909.474 |
|  |  | 2-Factor (WM with IR) | 53.375 | 19 | < 0.001 | 0.065 (0.045, 0.086) | 0.921 | 3874.764 | 3897.139 |
|  |  | 3-Factor^a,b,c,d^ | 32.176 | 17 | 0.014 | 0.045 (0.020, 0.069) | 0.965 | 3856.743 | 3880.908 |
|  | 4 | 1-Factor | 98.614 | 20 | < 0.001 | 0.098 (0.079, 0.118) | 0.777 | 3828.735 | 3848.966 |
|  |  | 2-Factor (IR with CM) | 54.483 | 19 | < 0.001 | 0.067 (0.047, 0.089) | 0.899 | 3787.066 | 3808.140 |
|  |  | 2-Factor (WM with CM) | 74.561 | 19 | < 0.001 | 0.084 (0.065, 0.105) | 0.842 | 3806.105 | 3827.179 |
|  |  | 2-Factor (WM with IR) | 65.114 | 19 | < 0.001 | 0.077 (0.057, 0.098) | 0.869 | 3798.073 | 3819.147 |
|  |  | 3-Factor^a,b,c,d^ | 20.982 | 17 | 0.227 | 0.024 (0.000, 0.053) | 0.989 | 3758.079 | 3780.839 |

*Note*. ^a^Model with highest CFI; ^b^Model with lowest RMSEA; ^c^Model with lowest AIC; ^d^Model with lowest BICc. CFI = comparative fit index; RMSEA = root mean square error of approximation; AIC = Akaike information criterion; BICc = sample-size adjusted Bayesian information criterion; IR = interference resolution; CM = context monitoring; WM = working memory.

Table S6. *Factor Loadings from 1-Factor Model of Executive Function*

|  | **3rd-4th Grade Cohort** | | | | **5th-6th Grade Cohort** | | | | **7th-8th Grade Cohort** | | | |
| --- | --- | --- | --- | --- | --- | --- | --- | --- | --- | --- | --- | --- |
| **Task** | **Time-**  **point 1** | **Time-**  **point 2** | **Time-**  **point 3** | **Time-**  **point 4** | **Time-**  **point 1** | **Time-**  **point 2** | **Time-**  **point 3** | **Time-**  **point 4** | **Time-**  **point 1** | **Time-**  **point 2** | **Time-**  **point 3** | **Time-**  **point 4** |
| *B. SPAN* | 0.393 | 0.453 | 0.376 | 0.407 | 0.364 | 0.289 | 0.306 | 0.288 | 0.376 | 0.361 | 0.420 | 0.257 |
| *F. SPAN* | 0.506 | 0.345 | 0.339 | 0.485 | 0.408 | 0.375 | 0.319 | 0.400 | 0.286 | 0.340 | 0.320 | 0.312 |
| *SUST ATTN* | 0.491 | 0.406 | 0.297 | 0.340 | 0.357 | 0.461 | 0.311 | 0.255 | 0.382 | 0.260 | 0.407 | 0.406 |
| *IMPUL ATTN* | 0.584 | 0.590 | 0.498 | 0.359 | 0.610 | 0.451 | 0.297 | 0.395 | 0.390 | 0.418 | 0.478 | 0.465 |
| *TAP & TRACE* | 0.615 | 0.581 | 0.402 | 0.501 | 0.491 | 0.517 | 0.314 | 0.482 | 0.441 | 0.554 | 0.512 | 0.484 |
| *STROOP* | 0.763 | 0.673 | 0.607 | 0.654 | 0.699 | 0.575 | 0.761 | 0.667 | 0.484 | 0.598 | 0.613 | 0.569 |
| *FLANKER* | 0.664 | 0.638 | 0.664 | 0.662 | 0.729 | 0.658 | 0.666 | 0.704 | 0.780 | 0.771 | 0.736 | 0.701 |
| *BOXED* | 0.380 | 0.307 | 0.441 | 0.437 | 0.350 | 0.441 | 0.609 | 0.582 | 0.397 | 0.528 | 0.407 | 0.387 |

*Note.* Factor loadings are standardized. B. SPAN = Backward Spatial Span; F. SPAN = Forward Spatial Span; SUST ATTN = Sustained Attention; IMPUL ATTN = Impulsive Attention.

Table S7. *Factor Loadings and Correlations from 2-Factor Model of Executive Function Combining Context Monitoring and Interference Resolution*

|  |  | **3rd-4th Grade Cohort** | | | | **5th-6th Grade Cohort** | | | | **7th-8th Grade Cohort** | | | |
| --- | --- | --- | --- | --- | --- | --- | --- | --- | --- | --- | --- | --- | --- |
| **Factor** | **Task** | **Time-**  **point 1** | **Time-**  **point 2** | **Time-**  **point 3** | **Time-**  **point 4** | **Time-**  **point 1** | **Time-**  **point 2** | **Time-**  **point 3** | **Time-**  **point 4** | **Time-**  **point 1** | **Time-**  **point 2** | **Time-**  **point 3** | **Time-**  **point 4** |
| Working Memory | *B. SPAN* | 0.529 | 0.613 | 0.373 | 0.483 | 0.530 | 0.459 | 0.509 | 0.427 | 0.581 | 0.537 | 0.672 | 0.528 |
|  | *F. SPAN* | 0.711 | 0.449 | 0.336 | 0.589 | 0.607 | 0.630 | 0.534 | 0.631 | 0.421 | 0.503 | 0.477 | 0.690 |
| Context Monitoring and Interference Resolution | *SUST ATTN* | 0.488 | 0.405 | 0.297 | 0.343 | 0.364 | 0.462 | 0.318 | 0.255 | 0.383 | 0.261 | 0.408 | 0.407 |
|  | *IMPUL ATTN* | 0.588 | 0.588 | 0.498 | 0.358 | 0.615 | 0.441 | 0.297 | 0.386 | 0.388 | 0.421 | 0.482 | 0.469 |
|  | *TAP & TRACE* | 0.616 | 0.577 | 0.402 | 0.509 | 0.490 | 0.518 | 0.315 | 0.485 | 0.442 | 0.552 | 0.517 | 0.481 |
|  | *STROOP* | 0.771 | 0.680 | 0.607 | 0.656 | 0.698 | 0.577 | 0.762 | 0.663 | 0.479 | 0.595 | 0.607 | 0.570 |
|  | *FLANKER* | 0.665 | 0.649 | 0.664 | 0.664 | 0.734 | 0.670 | 0.668 | 0.716 | 0.794 | 0.780 | 0.750 | 0.710 |
|  | *BOXED* | 0.387 | 0.312 | 0.441 | 0.442 | 0.350 | 0.450 | 0.611 | 0.584 | 0.397 | 0.529 | 0.409 | 0.390 |
| **Factor Correlation** | | | | | | | | | | | | | |
| *r*(WM, CM + IR) | | 0.676 | 0.706 | 1.011 | 0.787 | 0.632 | 0.550 | 0.558 | 0.599 | 0.609 | 0.630 | 0.583 | 0.396 |

*Note.* Factor loadings are standardized. WM = working memory; CM = context monitoring; IR = interference resolution; B. SPAN = Backward Spatial Span; F. SPAN = Forward Spatial Span; SUST ATTN = Sustained Attention; IMPUL ATTN = Impulsive Attention.

Table S8. *Factor Loadings and Correlations from 2-Factor Model of Executive Function Combining Working Memory and Context Monitoring*

|  |  | **3rd-4th Grade Cohort** | | | | **5th-6th Grade Cohort** | | | | **7th-8th Grade Cohort** | | | |
| --- | --- | --- | --- | --- | --- | --- | --- | --- | --- | --- | --- | --- | --- |
| **Factor** | **Task** | **Time**  **point 1** | **Time**  **point 2** | **Time**  **point 3** | **Time**  **point 4** | **Time**  **point 1** | **Time**  **point 2** | **Time**  **point 3** | **Time**  **point 4** | **Time**  **point 1** | **Time**  **point 2** | **Time**  **point 3** | **Time**  **point 4** |
| Working Memory + Context Monitoring | *B. SPAN* | 0.398 | 0.481 | 0.413 | 0.401 | 0.368 | 0.331 | 0.334 | 0.362 | 0.401 | 0.397 | 0.435 | 0.281 |
|  | *F. SPAN* | 0.509 | 0.377 | 0.349 | 0.522 | 0.420 | 0.425 | 0.366 | 0.397 | 0.300 | 0.362 | 0.329 | 0.295 |
|  | *SUST ATTN* | 0.496 | 0.445 | 0.314 | 0.384 | 0.368 | 0.488 | 0.348 | 0.336 | 0.401 | 0.295 | 0.465 | 0.497 |
|  | *IMPUL ATTN* | 0.587 | 0.655 | 0.565 | 0.430 | 0.634 | 0.527 | 0.371 | 0.549 | 0.423 | 0.460 | 0.539 | 0.548 |
|  | *TAP & TRACE* | 0.618 | 0.624 | 0.418 | 0.523 | 0.507 | 0.553 | 0.363 | 0.557 | 0.468 | 0.592 | 0.550 | 0.569 |
| Interference Resolution | *STROOP* | 0.769 | 0.764 | 0.611 | 0.667 | 0.707 | 0.608 | 0.772 | 0.668 | 0.478 | 0.597 | 0.616 | 0.598 |
|  | *FLANKER* | 0.666 | 0.710 | 0.698 | 0.696 | 0.741 | 0.707 | 0.664 | 0.732 | 0.811 | 0.787 | 0.773 | 0.756 |
|  | *BOXED* | 0.385 | 0.306 | 0.460 | 0.456 | 0.359 | 0.492 | 0.613 | 0.608 | 0.397 | 0.537 | 0.425 | 0.413 |
| **Factor Correlation** | | | | | | | | | | | | | |
| *r*(WM + CM, IR) | | 0.979 | 0.708 | 0.827 | 0.824 | 0.927 | 0.730 | 0.818 | 0.708 | 0.883 | 0.861 | 0.829 | 0.700 |

*Note.* Factor loadings are standardized. WM = working memory; CM = context monitoring; IR = interference resolution; B. SPAN = Backward Spatial Span; F. SPAN = Forward Spatial Span; SUST ATTN = Sustained Attention; IMPUL ATTN = Impulsive Attention.

Table S9. *Factor Loadings and Correlations from 2-Factor Model of Executive Function Combining Working Memory and Interference Resolution*

|  |  | **3rd-4th Grade Cohort** | | | | **5th-6th Grade Cohort** | | | | **7th-8th Grade Cohort** | | | |
| --- | --- | --- | --- | --- | --- | --- | --- | --- | --- | --- | --- | --- | --- |
| **Factor** | **Task** | **Time-**  **point 1** | **Time-**  **point 2** | **Time-**  **point 3** | **Time-**  **point 4** | **Time-**  **point 1** | **Time-**  **point 2** | **Time-**  **point 3** | **Time-**  **point 4** | **Time-**  **point 1** | **Time-**  **point 2** | **Time-**  **point 3** | **Time-**  **point 4** |
| Working Memory + Interference Resolution | *B. SPAN* | 0.394 | 0.433 | 0.372 | 0.425 | 0.372 | 0.282 | 0.308 | 0.281 | 0.375 | 0.360 | 0.431 | 0.249 |
|  | *F.SPAN* | 0.509 | 0.324 | 0.340 | 0.483 | 0.411 | 0.365 | 0.322 | 0.408 | 0.286 | 0.341 | 0.331 | 0.326 |
|  | *STROOP* | 0.501 | 0.463 | 0.344 | 0.442 | 0.398 | 0.514 | 0.531 | 0.367 | 0.461 | 0.332 | 0.509 | 0.523 |
|  | *FLANKER* | 0.594 | 0.679 | 0.560 | 0.521 | 0.681 | 0.497 | 0.496 | 0.547 | 0.469 | 0.509 | 0.602 | 0.591 |
|  | *BOXED* | 0.623 | 0.631 | 0.423 | 0.612 | 0.515 | 0.574 | 0.415 | 0.641 | 0.503 | 0.623 | 0.597 | 0.575 |
| Context Monitoring | *SUST ATTN* | 0.766 | 0.729 | 0.610 | 0.660 | 0.708 | 0.603 | 0.778 | 0.681 | 0.485 | 0.599 | 0.631 | 0.604 |
|  | *IMPUL ATTN* | 0.665 | 0.687 | 0.674 | 0.689 | 0.735 | 0.678 | 0.657 | 0.707 | 0.795 | 0.778 | 0.746 | 0.735 |
|  | *TAP & TRACE* | 0.383 | 0.306 | 0.451 | 0.443 | 0.360 | 0.467 | 0.613 | 0.605 | 0.399 | 0.534 | 0.422 | 0.414 |
| **Factor Correlation** | | | | | | | | | | | | | |
| *r*(WM + IR, CM) | | 0.970 | 0.758 | 0.857 | 0.660 | 0.862 | 0.802 | 0.557 | 0.642 | 0.786 | 0.794 | 0.728 | 0.651 |

*Note.* Factor loadings are standardized. WM = working memory; CM = context monitoring; IR = interference resolution; B. SPAN = Backward Spatial Span; F. SPAN = Forward Spatial Span; SUST ATTN = Sustained Attention; IMPUL ATTN = Impulsive Attention.

Table S10. *Factor Loadings and Correlations from 3-Factor Model of Executive Function*

|  |  | **3rd-4th Grade Cohort** | | | | **5th-6th Grade Cohort** | | | | **7th-8th Grade Cohort** | | | |
| --- | --- | --- | --- | --- | --- | --- | --- | --- | --- | --- | --- | --- | --- |
| **Factor** | **Task** | **Time-**  **point 1** | **Time-**  **point 2** | **Time-**  **point 3** | **Time-**  **point 4** | **Time-**  **point 1** | **Time-**  **point 2** | **Time-**  **point 3** | **Time-**  **point 4** | **Time-**  **point 1** | **Time-**  **point 2** | **Time-**  **point 3** | **Time-**  **point 4** |
| Working Memory | *B. SPAN* | 0.524 | 0.606 | 0.382 | 0.490 | 0.538 | 0.458 | 0.520 | 0.428 | 0.579 | 0.536 | 0.673 | 0.495 |
|  | *F.SPAN* | 0.717 | 0.453 | 0.327 | 0.580 | 0.599 | 0.631 | 0.522 | 0.631 | 0.422 | 0.504 | 0.476 | 0.736 |
| Context Monitoring | *SUST ATTN* | 0.495 | 0.458 | 0.322 | 0.445 | 0.402 | 0.500 | 0.563 | 0.367 | 0.465 | 0.333 | 0.506 | 0.525 |
|  | *IMPUL ATTN* | 0.597 | 0.680 | 0.580 | 0.525 | 0.682 | 0.517 | 0.473 | 0.550 | 0.466 | 0.512 | 0.603 | 0.593 |
|  | *TAP & TRACE* | 0.622 | 0.628 | 0.419 | 0.608 | 0.511 | 0.568 | 0.407 | 0.638 | 0.504 | 0.621 | 0.598 | 0.572 |
| Interference Resolution | *STROOP* | 0.771 | 0.761 | 0.612 | 0.665 | 0.706 | 0.601 | 0.774 | 0.675 | 0.478 | 0.594 | 0.618 | 0.600 |
|  | *FLANKER* | 0.664 | 0.713 | 0.697 | 0.702 | 0.742 | 0.713 | 0.661 | 0.723 | 0.812 | 0.791 | 0.772 | 0.755 |
|  | *BOXED* | 0.386 | 0.307 | 0.461 | 0.452 | 0.359 | 0.492 | 0.614 | 0.609 | 0.396 | 0.535 | 0.423 | 0.413 |
| **Factor Correlations** | | | | | | | | | | | | | |
| *r*(WM, CM) | | 0.640 | 0.726 | 1.049 | 0.570 | 0.537 | 0.613 | 0.172 | 0.437 | 0.447 | 0.510 | 0.435 | 0.249 |
| *r*(WM, IR) | | 0.687 | 0.574 | 0.918 | 0.786 | 0.647 | 0.448 | 0.580 | 0.591 | 0.613 | 0.624 | 0.592 | 0.390 |
| *r*(CM, IR) | | 0.984 | 0.690 | 0.797 | 0.644 | 0.873 | 0.748 | 0.573 | 0.631 | 0.788 | 0.792 | 0.734 | 0.647 |

*Note.* Factor loadings are standardized. WM = working memory; CM = context monitoring; IR = interference resolution; B. SPAN = Backward Spatial Span; F. SPAN = Forward Spatial Span; SUST ATTN = Sustained Attention; IMPUL ATTN = Impulsive Attention.

Table S11. *Comparison of* *Community Detection Results Between Observed and Re-sampled Data*

|  |  |  |  | **Most Frequent Solution** | | **2^nd^ Most Frequent Solution** | |
| --- | --- | --- | --- | --- | --- | --- | --- |
| **Cohort** | **Timepoint** | **Data Set** | **Unique Solutions** | **Community Organization** | **Freq (%)** | **Community Organization** | **Freq (%)** |
| *3^rd^-4^th^ grade cohort* | 1 | Observed | 6 | 1: B. SPAN, F. SPAN  2: SUST ATTN, IMPUL ATTN, TAP & TRACE, FLANKER, STROOP, BOXED | 90.4 | 1: B. SPAN, F. SPAN  2: SUST ATTN, IMPUL ATTN  3: TAP & TRACE, STROOP, FLANKER, BOXED | 5.0 |
|  |  | Resampled | 52 | 1: B. SPAN, F. SPAN  2: SUST ATTN, IMPUL ATTN, TAP & TRACE, FLANKER, STROOP, BOXED | 16.6 | 1: B. SPAN, F. SPAN  2: SUST ATTN, IMPUL ATTN  3: TAP & TRACE, STROOP, FLANKER, BOXED | 13.4 |
|  | 2 | Observed | 8 | 1: B. SPAN, F. SPAN, SUST ATTN, IMPUL ATTN, TAP & TRACE  2: STROOP, FLANKER, BOXED | 58.3 | 1: B. SPAN, F. SPAN  2: SUST ATTN, IMPUL ATTN, TAP & TRACE  3:FLANKER, STROOP  4: BOXED | 33.7 |
|  |  | Resampled | 35 | 1: B. SPAN, F. SPAN, SUST ATTN, IMPUL ATTN, TAP & TRACE  2: STROOP, FLANKER, BOXED | 24.2 | 1: B. SPAN, F. SPAN  2: SUST ATTN, IMPUL ATTN, TAP & TRACE, BOXED  3: STROOP, FLANKER | 22.2 |
|  | 3* | Observed | 18 | 1: B. SPAN, SUST ATTN, IMPUL ATTN, TAP & TRACE  2: F. SPAN, FLANKER, STROOP, BOXED | 49.8 | 1: B. SPAN, TAP & TRACE  2: SUST ATTN, IMPUL ATTN  3: F. SPAN, FLANKER, STROOP, BOXED | 24.4 |
|  |  | Resampled | 79 | 1: B. SPAN, SUST ATTN, IMPUL ATTN, BOXED  2: F. SPAN, TAP & TRACE, FLANKER, STROOP | 12.2 | 1: B. SPAN, F. SPAN, IMPUL ATTN, TAP & TRACE  2: SUST ATTN, STROOP, FLANKER, BOXED | 10.8 |
|  | 4 | Observed | 7 | 1: B. SPAN, F. SPAN, FLANKER, STROOP, BOXED  2: SUST ATTN, IMPUL ATTN, TAP & TRACE, | 93.2 | 1: B. SPAN, F. SPAN, FLANKER, STROOP  2: SUST ATTN, IMPUL ATTN, TAP & TRACE  3: BOXED | 2.6 |
|  |  | Resampled | 33 | 1: B. SPAN, F. SPAN, FLANKER, STROOP, BOXED  2: SUST ATTN, IMPUL ATTN, TAP & TRACE, | 30.4 | 1: B. SPAN, F. SPAN  2: SUST ATTN, IMPUL ATTN, TAP &  TRACE  3: FLANKER, STROOP, BOXED | 17.0 |
| *5^th^-6^th^ grade cohort* | 1 | Observed | 13 | 1: B. SPAN, F. SPAN  2: SUST ATTN, STROOP, BOXED  3: IMPUL ATTN, TAP & TRACE, FLANKER | 51.1 | 1: B. SPAN, F. SPAN  2: SUST ATTN, IMPUL ATTN  3: TAP & TRACE FLANKER, STROOP, BOXED | 20.8 |
|  |  | Resampled | 42 | 1: B. SPAN, F. SPAN  2: SUST ATTN, STROOP, BOXED  3: IMPUL ATTN, TAP & TRACE, FLANKER | 18.0 | 1: B. SPAN, F. SPAN  2: SUST ATTN, IMPUL ATTN, TAP & TRACE  3: FLANKER, STROOP, BOXED | 17.4 |
|  | 2 | Observed | 6 | 1: B. SPAN, F. SPAN  2: SUST ATTN, IMPUL ATTN, TAP & TRACE  3: FLANKER, STROOP, BOXED | 44.7 | 1: B. SPAN, F. SPAN, SUST ATTN, IMPUL ATTN, TAP & TRACE  2: FLANKER, STROOP, BOXED | 32.1 |
|  |  | Resampled | 19 | 1: B. SPAN, F. SPAN  2: SUST ATTN, IMPUL ATTN, TAP & TRACE  3: FLANKER, STROOP, BOXED | 29.8 | 1: B. SPAN, F. SPAN, IMPUL ATTN  2: SUST ATTN, TAP & TRACE, FLANKER, STROOP, BOXED | 21.0 |
|  | 3 | Observed | 9 | 1: B. SPAN, F. SPAN  2: SUST ATTN, IMPUL ATTN, TAP & TRACE  3: FLANKER, STROOP, BOXED | 93.1 | 1: B. SPAN, F. SPAN  2: SUST ATTN, IMPUL ATTN  3: TAP & TRACE  4: FLANKER, STROOP, BOXED | 5.2 |
|  |  | Resampled | 23 | 1: B. SPAN, F. SPAN  2: SUST ATTN, IMPUL ATTN, TAP & TRACE  3: FLANKER, STROOP, BOXED | 61.8 | 1: B. SPAN, F. SPAN, FLANKER, STROOP, BOXED  2: SUST ATTN, IMPUL ATTN, TAP & TRACE | 11.0 |
|  | 4 | Observed | 11 | 1: B. SPAN, F. SPAN  2: SUST ATTN, IMPUL ATTN, TAP & TRACE  3: FLANKER, STROOP, BOXED | 93.3 | 1: B. SPAN  2: F. SPAN  3: SUST ATTN, IMPUL ATTN, TAP & TRACE  4: FLANKER, STROOP, BOXED | 2.4 |
|  |  | Resampled | 17 | 1: B. SPAN, F. SPAN  2: SUST ATTN, IMPUL ATTN, TAP & TRACE  3: FLANKER, STROOP, BOXED | 55.4 | 1: B. SPAN, SUST ATTN, IMPUL ATTN, TAP & TRACE  2: F. SPAN, FLANKER, STROOP, BOXED | 24.4 |
|  |  |  |  |  |  |  |  |
|  |  |  |  |  |  |  |  |
|  |  |  |  |  |  |  |  |
|  |  |  |  |  |  |  |  |
| *7^th^-8^th^ grade cohort* | 1 | Observed | 13 | 1: B. SPAN, F. SPAN  2: SUST ATTN, IMPUL ATTN, TAP & TRACE  3: FLANKER, STROOP, BOXED | 95.8 | 1: B. SPAN, F. SPAN  2: SUST ATTN, IMPUL ATTN, TAP & TRACE, FLANKER  3: STROOP, BOXED | 1.3 |
|  |  | Resampled | 75 | 1: B. SPAN, F. SPAN  2: SUST ATTN, IMPUL ATTN, TAP & TRACE  3: FLANKER, STROOP, BOXED | 22.8 | 1: B. SPAN, F. SPAN, FLANKER, STROOP, BOXED  2: SUST ATTN, IMPUL ATTN, TAP & TRACE | 7.4 |
|  | 2 | Observed | 3 | 1: B. SPAN, F. SPAN  2: SUST ATTN, IMPUL ATTN  3: TAP & TRACE, FLANKER, STROOP, BOXED | 87.0 | 1: B. SPAN, F. SPAN  2: SUST ATTN, IMPUL ATTN, TAP & TRACE  3: FLANKER, STROOP, BOXED | 12.9 |
|  |  | Resampled | 42 | 1: B. SPAN, F. SPAN  2: SUST ATTN, IMPUL ATTN  3: TAP & TRACE, FLANKER, STROOP, BOXED | 38.0 | 1: B. SPAN, F. SPAN  2: SUST ATTN, IMPUL ATTN, TAP & TRACE  3: FLANKER, STROOP, BOXED | 26.4 |
|  | 3 | Observed | 6 | 1: B. SPAN, F. SPAN  2: SUST ATTN, IMPUL ATTN, TAP & TRACE  3: FLANKER, STROOP, BOXED | 95.1 | 1: B. SPAN, F. SPAN, STROOP  2: SUST ATTN, IMPUL ATTN, TAP & TRACE  3: FLANKER, BOXED | 1.7 |
|  |  | Resampled | 19 | 1: B. SPAN, F. SPAN  2: SUST ATTN, IMPUL ATTN, TAP & TRACE  3: FLANKER, STROOP, BOXED | 51.6 | 1: B. SPAN, F. SPAN, FLANKER, STROOP, BOXED  2: SUST ATTN, IMPUL ATTN, TAP & TRACE | 12.8 |
|  | 4 | Observed | 13 | 1: B. SPAN, F. SPAN  2: SUST ATTN, IMPUL ATTN, TAP & TRACE  3: FLANKER, STROOP, BOXED | 96.3 | 1: B. SPAN, F. SPAN  2: SUST ATTN, IMPUL ATTN, TAP & TRACE  3: FLANKER, STROOP  4: BOXED | 1.3 |
|  |  | Resampled | 11 | 1: B. SPAN, F. SPAN  2: SUST ATTN, IMPUL ATTN, TAP & TRACE  3: FLANKER, STROOP, BOXED | 91.8 | 1: B. SPAN, F. SPAN  2: SUST ATTN, IMPUL ATTN, TAP & TRACE, FLANKER  3: STROOP, BOXED | 3.4 |

*Note:* *Difference between most frequent solution in observed data and re-sampled data; B. SPAN = Backward Spatial Span; F. SPAN = Forward Spatial Span; SUST ATTN = Sustained Attention; IMPUL ATTN = Impulsive Attention.
